# Advanced Drug Carriers: A Review of Selected Protein, Polysaccharide, and Lipid Drug Delivery Platforms

**DOI:** 10.3390/ijms25020786

**Published:** 2024-01-08

**Authors:** Mateusz Jamroży, Sonia Kudłacik-Kramarczyk, Anna Drabczyk, Marcel Krzan

**Affiliations:** 1 Jerzy Haber Institute of Catalysis and Surface Chemistry, Polish Academy of Sciences, 8 Niezapominajek Str., 30-239 Krakow, Poland; mateusz.jamrozy@student.pk.edu.pl; 2 Department of Materials Engineering, Faculty of Materials Engineering and Physics, Cracow University of Technology, 37 Jana Pawła II Av., 31-864 Krakow, Poland; sonia.kudlacik-kramarczyk@pk.edu.pl (S.K.-K.); anna.drabczyk2@pk.edu.pl (A.D.)

**Keywords:** lipids, bionanocomposite, liposomes, solid lipid nanoparticles (SLNs), gelatin, cellulose, albumin

## Abstract

Studies on bionanocomposite drug carriers are a key area in the field of active substance delivery, introducing innovative approaches to improve drug therapy. Such drug carriers play a crucial role in enhancing the bioavailability of active substances, affecting therapy efficiency and precision. The targeted delivery of drugs to the targeted sites of action and minimization of toxicity to the body is becoming possible through the use of these advanced carriers. Recent research has focused on bionanocomposite structures based on biopolymers, including lipids, polysaccharides, and proteins. This review paper is focused on the description of lipid-containing nanocomposite carriers (including liposomes, lipid emulsions, lipid nanoparticles, solid lipid nanoparticles, and nanostructured lipid carriers), polysaccharide-containing nanocomposite carriers (including alginate and cellulose), and protein-containing nanocomposite carriers (e.g., gelatin and albumin). It was demonstrated in many investigations that such carriers show the ability to load therapeutic substances efficiently and precisely control drug release. They also demonstrated desirable biocompatibility, which is a promising sign for their potential application in drug therapy. The development of bionanocomposite drug carriers indicates a novel approach to improving drug delivery processes, which has the potential to contribute to significant advances in the field of pharmacology, improving therapeutic efficacy while minimizing side effects.

## 1. Introduction

In today’s world, developing modern therapies and strategies for delivering active substances requires innovative approaches [1,2] that balance therapeutic effectiveness with minimizing adverse effects. Traditional drug delivery methods include classical approaches such as oral, intravenous, dermal, or muscular administration of the active substance without the use of special carriers [3,4]. This works by directly delivering the active substance to the body, but has several significant problems. With oral delivery, drugs are prone to degradation in the gastrointestinal tract, which can lead to a loss of efficacy. In addition, active substances may be susceptible to rapid excretion from the body, reducing their blood concentration and therapeutic efficacy [5].

Traditional drug delivery methods are often characterized by a lack of selectivity, meaning that active substances spread throughout the body, affecting both diseased and healthy tissues or cells [6]. This phenomenon can lead to side effects and reduce the overall effectiveness of therapies. In contrast, drug carriers are an advanced tool, enabling the precise delivery of active substances where they are needed, representing a significant advancement in the therapeutic field. Drug carriers enable the targeted delivery of active substances. This phenomenon, known as targeting, is becoming a key element in improving treatment efficacy [7,8]. Drug-targeting approaches are based on precisely targeting drugs to disease areas, i.e., specific tissues or cells in the body, thus minimizing the impact on unaffected areas. These approaches lead to the accumulation of drugs in pathological sites, thereby increasing the effectiveness of the treatment. Targeted tissue delivery is often based on the area’s unique physical, chemical, or biological properties [9,10]. For example, in the case of cancerous tumors, carriers can be engineered to preferentially accumulate in the blood vessels surrounding the tumor (enhanced permeability and retention (EPR) effect), thus enabling the selective delivery of the drug to the tumor area [11]. For the treatment of neurological diseases, drug carriers can be designed to penetrate the blood–brain barrier and deliver active substances directly to the brain [12]. In turn, introducing receptors specific to the ligands on the surface of target cells allows for targeted delivery to the specific cells of a particular organ or tissue [13].

**Figure 1:**
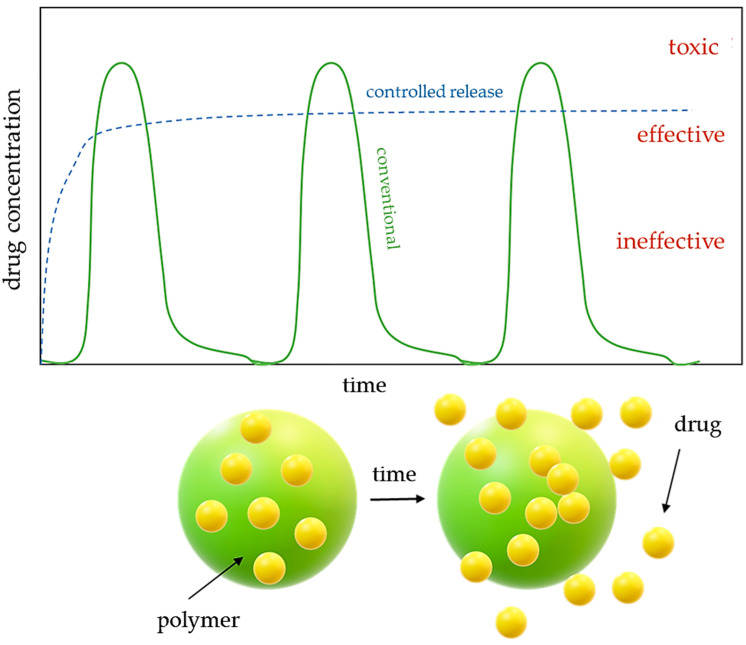
Procedure of controlled drug release from drug carriers (above: concentration of the drug released over time; below: drug release from the polymer matrix).

In addition, drug carriers allow for the controlled release of the active substances, which increases drug stability and minimizes loss of activity. Below (Figure 1), a scheme for the controlled release of active substances from drug carriers is presented. The multiple conventional drug dosages have been presented by the green circle line. During this delivery system, the drug portion is released immediately after its administration, which may have intense potential side effects and thus negatively affect the treatment. In the case of controlled drug release (blue dashed line), the active substance is released over a prolonged time period at a predetermined rate.

Many aspects are analyzed considering drug delivery approaches involving both conventional delivery and delivery using adequate carriers. In conventional delivery, drugs are spread in the body via the blood, which can lead to the uncontrolled distribution of active substances and their accumulation within healthy tissues or cells as well. In the context of drug carriers, efficient blood circulation is crucial, as it enables the precise delivery of the active substance to the targeted tissues [14,15]. Carriers of active substances are designed to move through the bloodstream in an optimized manner. The importance of the structure of drug carriers and their physicochemical properties is crucial to maintaining their stability in the blood, minimizing interactions with the immune system, and efficiently delivering drugs. Hence, they are designed with several key factors in mind. An important aspect is the carrier’s structure, which must be tailored to allow for efficient circulation in the blood. Proper size, shape, and surface functionalization affect the bioavailability of the carrier and its ability to avoid early removal from the body. Additionally, carriers must show physicochemical stability to maintain structural integrity during their transport through the bloodstream. This is important to ensure drug delivery efficiency and reduce potential side effects. Properties that minimize interactions with the immune system are also crucial. Carriers must avoid recognition by immune system cells, which can lead to the neutralization or elimination of carriers before reaching their target sites [16,17,18]. 

In traditional drug delivery, active substances are administered systemically into the body, often leading to their distribution in tissues and organs. In this approach, the targeting of substances to specific cells is limited, potentially increasing the risk of side effects and reducing therapeutic efficacy. In contrast, carrier-based drug delivery enables increased precision in cellular uptake. Carriers can facilitate cellular penetration, improving therapeutic efficacy. In addition, functionalizing carriers with receptors specific to ligands present on the surface of target cells increases uptake selectivity, minimizing the impact on healthy cells. As a result, carrier-mediated drug delivery focuses on increasing cellular uptake efficiency, resulting in more targeted and effective treatments with minimal side effects [19,20].

Compared to traditional delivery methods, drug carriers represent a novel approach, potentially revolutionary in improving the efficacy of therapies, especially in the context of cancer treatment [21]. Synthetic and natural polymers represent two different categories of materials used in drug carrier design. Synthetic polymers, such as poly(acrylic acid) or poly(ethylene glycol), have a controlled chemical structure, making fine-tuning their physicochemical properties possible. On the other hand, natural polymers, such as cellulose or albumin, are derived from natural sources and often exhibit better biocompatibility. A similarity is the ability of both types of polymers to form carriers with controlled release of the active substance. However, natural polymers often exhibit better biodegradability and less toxicity, which can be beneficial in terms of eliminating their potential side effects [22,23].

Natural-derived polymers exhibit several important properties that provide advantages over synthetic polymers in drug carrier design:

- Biocompatibility: natural polymers are often natural components of the body (e.g., hyaluronic acid), which minimizes the risk of immune reactions and provides better biocompatibility compared to synthetic polymers.
- Biodegradability: most natural polymers are naturally degradable in the body, eliminating the need for surgical removal after treatment, which is particularly important, especially in terms of minimizing side effects and body burden.
- Diverse sources: polymers of natural origin, such as proteins, polysaccharides, or nucleic acids, can be obtained from a variety of sources, making it possible to tailor their properties to specific applications.
- Significant impact on biological interactions: natural polymers often exhibit the ability to interact with cells and tissues in the body, which can be used to increase the selectivity of drug carriers and facilitate cellular uptake [24,25,26].

As a result, the properties of natural polymers make them an attractive choice for drug carrier design due to their naturalness, biocompatibility, and potential to minimize negative effects on the patient’s body.

One of the areas of intense research focused on improving therapeutic efficiency is the field of bionanocomposites, which serve as advanced drug carriers by combining attractive features of nanomaterials with materials of natural origin [27,28]. 

Nanocomposites represent an intriguing combination of diverse materials, such as fats, proteins, and polysaccharides, forming comprehensive structures with nanometric dimensions. This unique class of materials has found its application as drug carriers, enabling the efficient delivery of active substances within the body [29,30,31,32]. In a therapeutic context, nanocomposites exhibit a number of promising features, such as stability, controlled drug release, and the ability to deliver active substances in a targeted manner [33,34]. 

Drug carriers based on nanocomposites utilizing biopolymers, such as polysaccharides, constitute a fascinating research area. These natural polymers, including chitosan [35] and cellulose [36], are characterized by biocompatibility and biodegradability, making them attractive candidates for medical applications. Additionally, nanocomposites can be tailored by introducing nanoadditives in the form of fats, opening up new perspectives for effectively transporting lipophilic substances [37].

Contemporary approaches to therapy and the delivery of active substances are gaining significance in the context of minimizing side effects while increasing therapeutic efficacy [38]. The development of modern therapies requires innovative strategies, and one of the areas of intense research is the field of biocomposites and bionanocomposites, which serve as advanced drug carriers [39]. Research on this topic is fundamental, drawing attention to the emerging applications of composites and nanocomposites as drug carriers. Their unique characteristics make them comprehensive structures. This particular class of materials finds practical application in efficiently delivering active substances within the body. Hence, the main goal was to characterize the latest achievements in the field of development of biocomposites and bionanocomposites as carriers of active substances. The analysis of the newest literature underscores the importance of this topic, especially in the context of improving the effectiveness of treatments. Additionally, conventional drug delivery methods contribute to the distribution of drugs within the whole body, while the delivery of active substances via the adequate carriers may lead to their accumulation mostly within the affected site, thereby reducing occurring side effects (accompanying among other treatments of cancer using cytostatic drugs) [40,41,42].

## 2. Lipid-Containing Carriers

### 2.1. Lipids—Short Characteristics

Lipids, also known as fats, constitute a group of compounds with diverse structures but similar physicochemical properties. They are insoluble in water but readily dissolve in organic solvents [43]. Lipids are categorized into several main groups, including simple fats, encompassing true fats (glycerol esters and higher fatty acids) and waxes (esters of higher monohydroxy alcohols and higher fatty acids). Other groups of lipids are complex fats, such as phospholipids and glycolipids, as well as fat-like compounds like sterols, carotenoids, chlorophylls, fat-soluble vitamins, and others [44]. Lipids play numerous crucial biological roles, serving as fundamental components of cell membranes, acting as an energy storage reservoir, providing protective functions (in the case of waxes), regulating differentiation and growth processes, and contributing to metabolic regulation.

**Figure 2:**
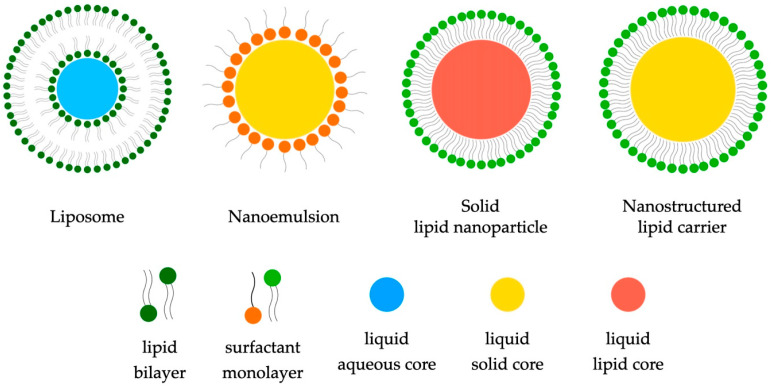
Sample lipid structures in biomedical applications.

The utilization of lipids in nanocomposites represents an innovative approach to efficiently deliver active substances. Leveraging their unique properties, lipids are pivotal in creating nanocomposites that effectively transport active substances. These bionanocomposites employ lipid carriers to encapsulate and protect active substances, enabling precise and controlled release. This approach improves the stability and bioavailability of active compounds, positively impacting therapeutic efficacy. The advanced use of lipids in the development of bionanocomposites opens new perspectives in the field of active substance delivery, with potential applications in various areas such as medicine, pharmacology, and cosmetics [45]. Lipids, constituting a diverse group of compounds with similar physicochemical properties, encompass various structures, including liposomes, nanoemulsions, solid lipid nanoparticles (SLNs), and nanostructured lipid carriers (NLCs) (Figure 2). 

Examples of such lipid utilization are presented in the following subsection.

#### 2.1.1. Liposomes

Liposomes are vesicular structures that are mainly composed of one or, sometimes, several aqueous compartments separated by closed concentric lipid bilayers, both natural and/or synthetic [46,47]. Molecules such as proteins, enzymes, chemotherapeutic drugs, nucleic acids, and imaging probes can be encapsulated inside these vesicles (for hydrophilic drugs), embedded within the bilayer (for hydrophobic drugs), or occasionally attached to the bilayer surface [48,49].

**Figure 3:**
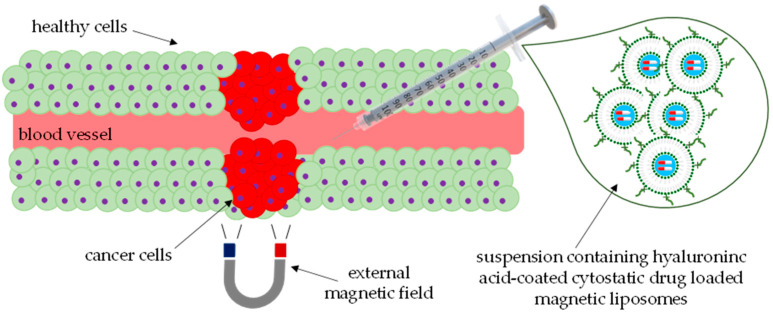
Scheme showing targeted drug delivery by means of magnetoliposomal nanocomposites.

Liposomes with modern chemical and physical properties exhibit enormous potential as drug carriers in cancer therapy. The team led by Amiri et al. [50] focused on developing an innovative electromagnetic drug delivery system, allowing the transfer of the anti-cancer drug imatinib (IM) by loading the active substance into liposomes containing magnetic nanocomposites. The goal was to achieve targeted drug delivery in the presence of an alternate magnetic field (AMF) to shorten the administration time, reduce the drug dosage, and minimize potential side effects. The scheme of this developed carrier is presented in Figure 3.

Drug targeting was possible due to the magnetic properties of the formulated carriers. Ultramicroscopic ZnFe_2_O_4_ nanoparticles with a distinctive coral shape and a diameter of 22.36 ± 2.21 nm were successfully synthesized in the presence of *Teucrium polium* using the hydrothermal method (green synthesis). Biocompatibility studies using the MTT test on the U87 cell line confirmed their safety. In vitro study results showed that the AMF significantly increased the release of IM from magnetoliposomal nanocomposites due to nanoparticle movement within the liposome structure at the applied frequency, affecting the bilayer’s permeability. Furthermore, in vivo biodistribution results suggested that the controlled magnetic accumulation of liposomes in target areas is faster and more efficient. This approach opens up new perspectives in the field of controlled drug release using magnetoliposomal nanocomposites, enhancing therapeutic efficiency while minimizing potential side effects.

Another research group, led by Ding et al. [51], conducted experiments on nanocomposite membranes containing liposomes. This team developed an innovative wound dressing to accelerate the healing process of wounds in diabetic patients. In the first stage of this study, a liposome with taxifolin (TL) was developed. Then, liposomes with taxifolin were combined with poly(vinyl alcohol) (PVA) and chitosan (CS) using electrostatic spinning to obtain nanocomposite membranes. Finally, studies were conducted on the mechanism of action of nanocomposite membranes in accelerating the healing of diabetic wounds. The nanocomposite membrane’s average diameter with poly(vinyl alcohol)/chitosan (PVA/CS/TL) containing TL was 429.43 ± 78.07 nm. In vitro experiment results showed that PVA/CS/TL membranes exhibited better water absorption, water vapor transmission rate (WVTR), porosity, hydrophilicity, mechanical, slow-release, antioxidant, and antibacterial properties. In vivo experiments confirmed that the wound healing rate in mice treated with PVA/CS/TL membranes for eighteen days was 98.39 ± 0.34%. Histopathological studies, immunohistochemical staining, and Western blot experiments also demonstrated that PVA/CS/TL membranes could promote wound healing in diabetic mice by inhibiting the activation of the inhibitor kappa B alpha (IκBα)/nuclear factor-kappa B (NF-κB) signaling pathway and related pro-inflammatory factors, leading to increased CD31 and VEGF expression in skin tissues.

Lu et al. [52] created HPCD@Lip nanocomposites, which were hydroxypropyl-β-cyclodextrin complexes enclosed in liposomes, to deliver dexamethasone effectively. To assess the integrity of these nanocomposites after passing through the conjunctival epithelial cell layer (HConEpiC) and eye tissues, Förster resonance energy transfer with near-infrared fluorescent dyes was applied, along with in vivo imaging. The structural integrity of the internal HPCD complexes was observed for the first time. Their results suggested that 23.1 ± 6.4% of nanocomposites and 41.2 ± 4.3% of HPCD complexes could pass through the HConEpiC layer intact after 1 h. Additionally, 15.3 ± 8.4% of intact nanocomposites were able to reach the sclera, and 22.9 ± 1.2% of intact HPCD complexes were able to reach the choroid/retina after 60 min in vivo, confirming that the dual carrier drug delivery system can effectively transport intact cyclodextrin complexes into the posterior segment of the eye.

Yu and colleagues [53] researched a gelatin hydrogel containing liposomes and methacrylate. They developed a drug delivery system capable of controlling the release of stromal cell-derived factor-1α (SDF-1α) from the stromal cells to stimulate mesenchymal stem cell migration. To protect the protein payload from hydrolytic degradation and control its release, SDF-1α was placed in anionic liposomes (lipoSDF), which were then embedded in gelatin methacrylate (GelMA), creating a nanocomposite hydrogel. Finally, the system’s ability to activate intracellular signaling in MSCs by analyzing the phosphorylation of key proteins in the mTOR pathway using Western blotting was assessed. This is the first study of its kind describing the delivery of liposomal SDF-1α using a nanocomposite approach.

Zhao et al. [54] created a liposome@AgAu nanocomposite for drug delivery, which can control drug release through near-infrared laser irradiation. Additionally, it enabled the monitoring of drug molecules using surface-enhanced Raman scattering (SERS) and fluorescent signals during the release process. Liposome@AgAu core/shell nanocomposites, prepared through galvanic exchange reactions (GRRs), exhibited regulated localized surface plasmon resonance (LSPR) absorption peaks from the visible to near-infrared region and high biocompatibility. Compared to pure doxorubicin (DOX) particles, liposome@AgAu nanocomposites with DOX showed lower cell toxicity in the MTT test. After loading DOX into liposome@AgAu, the fluorescence signal of DOX disappeared due to the resonance energy transfer from DOX to the metal shell. In contrast, the SERS signal of DOX in liposome@AgAu was significantly enhanced. Moreover, the liposome@AgAu nanocomposite demonstrated photothermal conversion ability under resonant laser radiation. Upon irradiation with a 633-nm wavelength laser, liposome@AgAu nanocomposites with DOX may release drug molecules to eliminate cancer cells. The fluorescent signal from DOX appeared after the release of the drug from liposome@AgAu, while the SERS signal was not visible. Thus, this nanocomposite can serve as a platform for photothermally controlled drug release and optical monitoring of the signal for drug molecules.

An interesting solution has been additionally investigated by Zhang et al. [55]. Here, multifunctional liposomal carriers of doxorubicin composed of fullerene and magnetic iron oxide nanoparticles combined with poly(ethylene glycol) have been developed. The release of cytostatic drugs was triggered via fullerene radiofrequency, while drug targeting by means of an external magnetic field was possible due to the presence of magnetic nanoparticles within the formulated carriers. In another work [56], the liposome surface has been functionalized using folic acid to enable the drug doxorubicin to target. The drug and, additionally, gold nanorods were inside the liposome carriers. Based on the performed research, it was concluded that functionalized carriers demonstrated higher cellular uptake than liposomes that have not been treated with folic acid. Moreover, it was also stated that the formulated materials showed toxicity towards cancer cells both in in vitro and in vivo investigations.

The table below presents a collection of solutions developed by researchers utilizing the properties of liposomes to obtain nanocomposites with biomedical properties (Table 1).

In this section, innovative approaches utilizing lipids in the development of bionanocomposites for the efficient delivery of active substances have been presented. Research analyses have focused on liposomes playing a pivotal role as drug carriers in anti-cancer therapy, diabetic wound healing, and controlled release of dexamethasone.

Currently performed studies aim to develop advanced drug delivery systems, capitalizing on the unique properties of liposomes. In the case of electromagnetic drug delivery, magnetic nanocomposites within liposomes enable the precise and controlled release of anti-cancer substances. Meanwhile, nanocomposite membranes with liposomes effectively accelerate diabetic wound healing. HPCD@Lip and liposome@AgAu nanocomposites also showcase advanced controlled release mechanisms, such as photothermal conversion and surface-enhanced Raman scattering.

The conclusions that have been drawn so far from these conducted studies indicate that using liposomes as carriers in nanocomposites opens new perspectives in active substance delivery, with potential applications in medicine, pharmacology, and cosmetology. Advanced technologies employing lipids can significantly enhance active substances’ stability, bioavailability, and therapeutic efficacy, representing a key direction in the development of modern therapies.

Developing liposome-based drug carriers is a promising approach to improving therapeutic efficacy, but at the same time, it brings a number of potential problems and challenges. Liposomes can be susceptible to destabilization because of storage conditions, leading to the loss of their structural integrity and reduced drug delivery efficiency. In addition, it can be problematic for liposomes to remain stable in the presence of digestive enzymes or other biological agents, which affects their persistence in the body [57,58]. Another aspect that requires further research is the control of the release of the active substance from liposomes, as this may be critical to achieving an adequate pharmacokinetic profile [59,60]. Additionally, there is a need to understand the interaction of liposomes with the immune system to avoid potential immune reactions [61,62]. Therefore, an important aspect in the design of liposome-based drug carriers is to pay attention to the storage conditions of these materials. They have a significant impact on the stability of liposomes, and this, in turn, is important in terms of maintaining their structural integrity and the efficiency of drug delivery. 

In the area of chemistry of obtaining and designing liposomes, a key issue is the precise adjustment of the chemical composition of lipids for therapeutic purposes. The optimal choice of the type of phospholipid and their proportions has a significant impact on the ability of liposomes to efficiently carry drugs. Controlling the size of liposomes during the preparation process is key to ensuring their stability and efficiency in carrying the active substance [63,64,65]. 

Understanding the chemical interactions between liposomes, the drug, and the biological environment is the foundation for effective drug carrier design. Considering these challenges, further research is needed to improve the stability, release control, and biological interactions of liposomes. A definitive understanding of these aspects is crucial for the successful implementation of liposomes as drug carriers, which requires advanced research and a long-term commitment to scientific research.

#### 2.1.2. Lipid Nanoemulsions

Stable nanoscale emulsion systems consisting of oil, water, and emulsifiers are utilized to enhance the solubility of lipophilic substances, finding applications in drug and nutrient delivery [66,67,68]. Andretto et al. [69] created nanocomposites using nanoemulsions embedded in alginate beads as an innovative solution to prolong the retention of nanoparticles in the gastrointestinal tract. They applied bioadhesive matrices based on microflows to protect the drug payload, including tofacitinib—an anti-inflammatory inhibitor. Nanoemulsions of approximately 110 nm were constructed to encapsulate this hydrophobic drug, effectively internalizing intestinal cells and delivering tofacitinib into macrophage cells, resulting in a reduced inflammatory response. Subsequently, nanoemulsions were placed in alginate microbeads with a size of 300 μm, forming a long-term stable pharmaceutical system. Ex vivo experiments on rat intestinal segments confirmed the bioadhesive ability of nanoemulsions compared to free nanoemulsions, emphasizing the benefits this hybrid system could bring to gastrointestinal pathology treatment.

Next, Hinger et al. [70], intrigued by the potential of lipid nanoemulsions containing m-tetrahydroxyphenylchlorin, conducted experiments on multicellular tumor spheroids of two different lipid sizes (50 nm and 120 nm) to assess their photodynamic effectiveness. Their emulsion production process involved mixing separately prepared aqueous phases containing the MyrjS40 surfactant dissolved in phosphate buffer (PBS) and a lipid phase consisting of soybean oil and wax (Suppocire NB) in a dissolved state. Their study confirmed that mTHPC (temoporfin) encapsulation delayed intracellular accumulation kinetics. Nevertheless, activated mTHPC trapped in 50-nm particles showed effective destruction of tumor spheroids as a free drug. Their analysis of cell death and gene expression provided evidence that encapsulation can lead to different mechanisms of cell elimination in photodynamic therapy (PDT).

In another interesting and alternative approach, Samadi et al. [71] explored strategies to overcome the limitations of quercetin (QC) in cancer therapy, using a hydrogel nanocomposite with agarose (AG)–polyvinylpyrrolidone (PVP)–hydroxyapatite (HAp) enclosed in a nanoemulsion. Despite the favorable characteristics of quercetin in cancer treatment, such as its low solubility, poor permeability, and short biological half-life, there are challenges in its practical application. These researchers aimed to increase the loading efficiency and simultaneously extend the period of quercetin release. The introduction of HAp nanopowders into the AG–PVP hydrogel resulted in improved loading efficiencies up to 61%. Interactions between nanopowders, the drug, and hydrogel polymers made the nanocomposite responsive to pH changes under acidic conditions, simultaneously controlling rapid release under neutral conditions. Subsequently, the loaded hydrogel with QC was placed in an aqueous-in-oil nanoemulsion, further extending the drug release time. pH-dependent QC release with prolonged effects for over 96 h was observed. According to the Korsmeyer–Peppas mathematical model, the release mechanism was atypical (diffusion controlled) at pH = 7.4 and atypical transport (dissolution controlled) at pH = 5.4. FTIR analysis confirmed the presence of all nanocomposite components, and the XRD results confirmed the incorporation of QC into the formed nanocomposite. This developed drug delivery system demonstrated its potential for further biomedical applications.

Continuing in the theme of overcoming limitations for quercetin, another research team, Ahmadi et al. [72], created an innovative hydrogel nanocomposite containing zinc oxide nanoparticles (ZnONPs), agarose, and poly(acrylic acid) (PAA) for quercetin (QC) delivery. Spherical-shaped nanocarriers were obtained through an ecological and simple double-emulsion method, where PAA/Aga/ZnONPs coated with the SPAN 80 surfactant were introduced into the hydrophobic olive oil phase. Characterization of the nanoemulsion using various techniques allowed for assessing the influence of the ZnONPs on the pH properties of the PAA/Aga hydrogel, creating a new platform for direct QC delivery. FTIR and XRD analyses confirmed the presence of all nanocomposite components in the final formulation. FE–SEM microscopic images revealed the nanocarriers’ spherical shape and surface uniformity, and zeta potential measurements confirmed their colloidal stability. The addition of ZnONPs increased the drug loading efficiency from 41.25% to 47.50% and the encapsulation efficiency from 83.0% to 87.25%. Slow drug release was observed at pH = 7.4 within less than 96 h, confirming the pH sensitivity of the nanoemulsion. The drug-release data at pH = 5.4 matched well with the first-order equation, while at pH = 7.4, it was better described with the Korsmeyer–Peppas model. Reduction in viable MCF7 cells in the presence of PAA/Aga/ZnONPs compared to PAA/Aga and the control sample indicated in vitro cytotoxicity of ZnONPs. The number of cells under late apoptosis in PAA/Aga/ZnONPs/QC (37.55%) was higher than in other formulations (ZnONPs, PAA/Aga, and PAA/Aga/ZnONPs), suggesting controlled and slower QC release from PAA/Aga/ZnONPs/QC. Thus, these ecological, biocompatible, and biodegradable nanoemulsions showed significant potential as carriers for QC with controlled release in breast cancer treatment.

Moreover, ongoing intense scientific research explores combinations like polyvinylpyrrolidone (PVP)/carboxymethyl cellulose (CMC)/γ-alumina with 5-fluorouracil (5-FU) to enhance the effectiveness of the cytostatic drug and limit its destructive impact on the body. Such studies were conducted by Shamsabadipour et al. [73].

Developing drug carriers based on lipid nanoemulsions offers promising prospects but also carries potential challenges that require further research. Nanoemulsions can be susceptible to destabilization associated with storage conditions, which affects their structural integrity and ability to effectively deliver drugs [74,75]. Controlling particle size and distribution in nanoemulsions is a key aspect that affects their stability and bioavailability [76]. In addition, the interactions of nanoemulsions with plasma proteins and cells can pose potential challenges, affecting drug delivery efficiency and possible immune reactions [77]. Thus, when designing drug carriers based on lipid nanoemulsions, special attention should be paid to selecting the reactants and reaction environment in such a way as to ensure adequate particle distribution and, thus, nanoemulsion stability. In addition, it is important to ensure adequate storage conditions to ensure the structural integrity of the nanoemulsion, as well as the ability to deliver drugs efficiently. 

In the chemistry of obtaining and designing drug carriers based on lipid nanoemulsions, a key aspect is the precise adjustment of the chemical composition of the lipids to the intended therapeutic targets. The adequate selection of the type of lipids and emulsifiers has a decisive impact on the stability and ability of the carriers to efficiently carry drugs. Controlling the particle size of nanoemulsions during the preparation process is key to ensuring their stability and efficiency in releasing the active substance [78,79]. Knowledge concerning the interactions between lipids and the active substance as well as the biological environment is crucial for the effective design of lipid nanoemulsion-based drug carriers. Further research is therefore needed to understand and counteract these issues and improve the parameters of nanoemulsions to maximize their effectiveness as drug carriers. Systematic experimentation and analyses are essential to fully realize the potential of lipid nanoemulsions in the field of drug delivery.

#### 2.1.3. Solid Lipid Nanoparticles (SLNs) and Nanostructured Lipid Carriers (NLCs)

**Figure 4:**
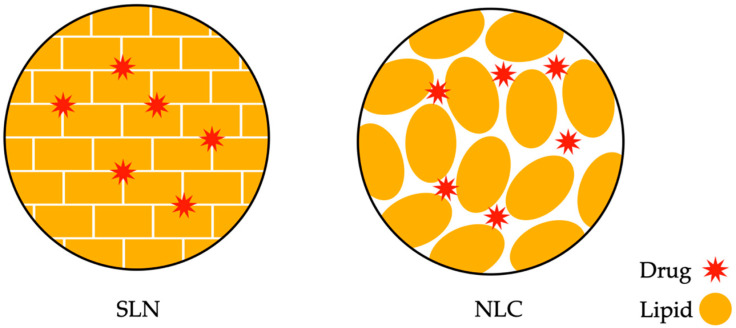
Comparison of the structures of solid lipid nanoparticles (SLNs) and nanostructured lipid carriers (NLCs) in terms of drug interaction reveals distinctive features in their design and functionality.

Another compound example is solid lipid nanoparticles (SLNs), which represent an advanced drug delivery form where the therapeutic substance is enclosed in a solid lipid core with nanometric dimensions. These nanoparticles are characterized by stability, controlled drug release, and their ability to enhance the bioavailability of the active substance. They constitute a promising platform for effective drug therapy [80,81]. On the other hand, nanostructured lipid carriers (NLCs) are advanced drug carriers that represent a developed version of lipid drug carriers. NLCs consist of lipids with a complex structure, including both liquid and solid oils, allowing for a more stable encapsulation of the active substance. Due to their nanostructural form, NLCs exhibit improved drug transport and release capabilities, making them a promising tool for delivering active substances, especially in the pharmaceutical context [82,83]. Both mentioned structures, i.e., SLNs and NLCs, are presented below in Figure 4.

One example of the application of solid lipid nanoparticles (SLN) in nanocomposites has been demonstrated in the research conducted by Vigani et al. [84]. Their study focused on the use of a composite nanosystem (CN) for local glioblastoma treatment, employing chitosan-coated solid lipid nanoparticles (c-SLNs) embedded in nanofibers of O-carboxymethyl chitosan (O-CMCS). Coacervation prepared solid lipid nanoparticles with stearic acid (SA-SLN) and behenic acid (BA-SLN). BA-SLN, containing 0.75% sodium salt of behenic acid and 3% poly(vinyl alcohol) (PVA), was selected for further investigation due to its small size. Subsequently, BA-SLN was coated with chitosan (CS), enhancing their accumulation in glioblastoma cells (U-373) after 6 h compared to the uncoated ones. The obtained c-BA-SLNs were then dissolved in polymer solutions and subjected to electrospinning, resulting in nanofibers with a diameter of 850 nm. Upon their dissolution in water, c-BA-SLNs retained their properties and zeta potential.

On the other hand, research on nanostructured lipid carriers (NLCs) was conducted by Shu et al.’s group [85]. They investigated nanostructured lipid carriers (NLCs) as drug carriers, synthesizing a nanoemulsion (NE) stabilized with rhamnolipid/chitosan, solid lipid nanoparticles (SLNs), and incorporating these structures into a composite hydrogel of κ-carrageenan/konjac glucomannan (KC-KGM). Their aim was to examine the impact of the lipid composition on the properties and performance of lutein-filled hydrogels (FHs). The use of solid lipids increased the viscosity and crystallinity of the emulsified lipids, affecting the rheological and textural properties of the FHs. NLC-FH showed the shortest delay time and the highest regeneration coefficient, confirming that filling the NLCs effectively improved the rheological properties of the FHs. Adding EGCG to NE/NLC/SLN-FH extended the half-life of lutein, and its stability increased, especially in NLC-FH with EGCG. During in vitro digestion, the FH delayed the release of lutein in the early stages, and EGCG increased the release of lutein in intestinal digestion, with the highest lutein bioavailability in NE-FH (29.67%) and NLC-FH (28.22%). These results suggest the potential application of rhamnolipid-stabilized lipids in hybrid gel systems to improve the properties and delivery of lipophilic compounds.

The development of drug carriers based on solid lipid nanoparticles (SLNs) and nanostructured lipid carriers (NLCs) brings the prospects of revolutionary solutions behind them, but at the same time, it generates potential challenges that require detailed research. In the case of the SLNs, their stability can be an issue due to the possible presence of a crystalline phase that affects the carriers’ ability to efficiently store and deliver drugs [86,87]. In the case of the NLCs, on the other hand, control of the morphology of the lipid structure and its effect on the release of the active substance is critical to achieving optimal therapeutic efficacy [88]. In addition, controlling the particle size and stability can be an issue in both cases, directly affecting the drug’s bioavailability and distribution in the body. 

In the context of the chemistry of obtaining and designing solid lipid nanoparticles (SLNs) and nano-structured lipid carriers (NLCs), it is crucial to fine-tune the chemical composition of lipids for therapeutic purposes. An appropriate selection of lipids and surface stabilizers is essential for the controlled synthesis of these structures. Thus, to sum up, advanced drug carriers, such as solid lipid nanoparticles (SLNs) and nanostructured lipid carriers (NLCs), represent promising strategies for delivering active substances, offering stability, controlled drug release, and the ability to improve bioavailability [89,90].

**Figure 5:**
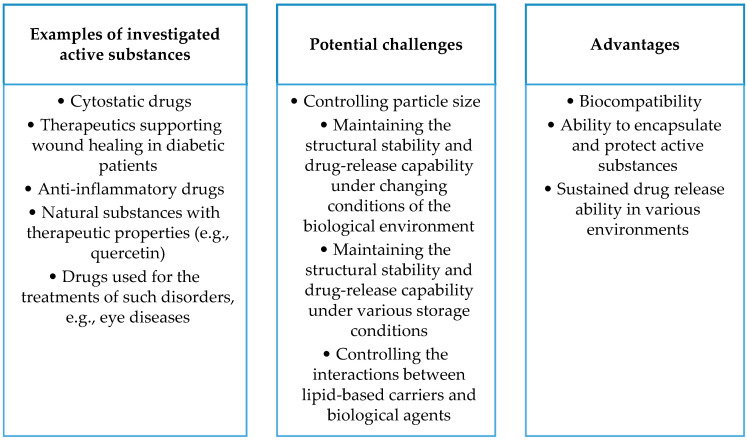
Summary of selected aspects of lipid-based drug carriers.

The figure below (Figure 5) compiles the advantages of lipid-based carriers (including liposomes, lipid nanoemulsions, solid lipid nanoparticles, and nano-structured lipid carriers) and the challenges accompanying the development of these carriers, as well as examples of active substances being tested.

There are a lot of challenges that should be widely considered during the preparation of lipid-based carriers of active substances. However, lipids definitely constitute promising compounds for further investigations within this area. Polysaccharides are another substance showing high potential in carrier design. The next section of this paper has characterized two types of them, i.e., alginates and cellulose, in terms of this application.

## 3. Polysaccharide-Based Nanocomposites

### 3.1. Alginate-Based Nanocomposites

**Figure 6:**
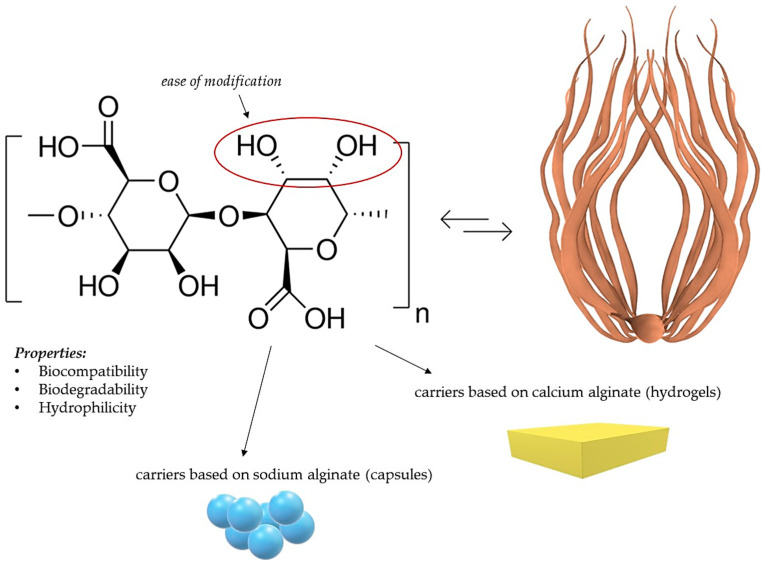
Alginic acid—formula, properties, and the process of obtaining it.

Alginic acid is an anionic polysaccharide obtained from brown algae, mainly consisting of glucuronic and mannuronic acid residues. It shows biocompatibility, biodegradability, and hydrophilicity. Many carboxyl groups in the structure of alginic acid make this compound easily modifiable [91,92]. The structural formula of alginic acid and its basic properties, as well as the preparation scheme, are presented in Figure 6.

Among the derivatives of alginic acid, particular interest is directed towards its salts (for example, calcium alginate or sodium alginate), which are widely used in many areas, including in drug delivery systems [93].

Sodium alginate is a biodegradable, biocompatible, hydrophobic, and water-soluble polysaccharide. It is also non-immunogenic and, importantly, shows chelating capability. Moreover, due to its ability to gel, alginate can be used to create matrices in which drugs are incorporated and then released at a specific rate, which is helpful for topical therapy and drug delivery [94,95]. It also shows good availability and low production costs, further supporting its drug carrier development [96]. Many works have widely discussed the potential of alginate-based materials in effectively delivering active substances [97,98,99,100].

Studies on developing drug delivery systems based on sodium alginate have been presented, for example, by Venkatasubbu et al. [101]. Here, sodium alginate-based composites were incorporated with hydroxyapatite (HAp) nanoparticles loaded previously with ciprofloxacin hydrochloride. This active substance shows antibacterial activity towards both Gram-negative and Gram-positive bacteria. The release investigations included comparing the release of the drug from hydroxyapatite nanoparticles incorporated into the alginate-based carrier and not introduced into any carrier. As a result of these experiments, it was demonstrated that the incorporation of drug-loaded HAp nanoparticles into other carriers resulted in the release of 15% less active substances. Hence, it may be concluded that these developed carriers ensure sustained drug release, providing high treatment efficacy and limiting the development of bacterial resistance. The application of an alginate-based nanocomposite as the drug carrier showing antibacterial activity was also investigated by Soumia et al. [102]. This work used magnetic nanoparticles (Fe_3_O_4_) and alginate to obtain nanocomposites to deliver amoxycillin. The release studies were both conducted in a simulated gastric medium (pH = 2.1) and biolysis serum (pH = 7.0). It was demonstrated that significantly more active substances were released in the environment with a neutral pH. Notably, the developed nanocomposites also showed antibacterial properties towards the tested bacterial strains. 

**Figure 7:**
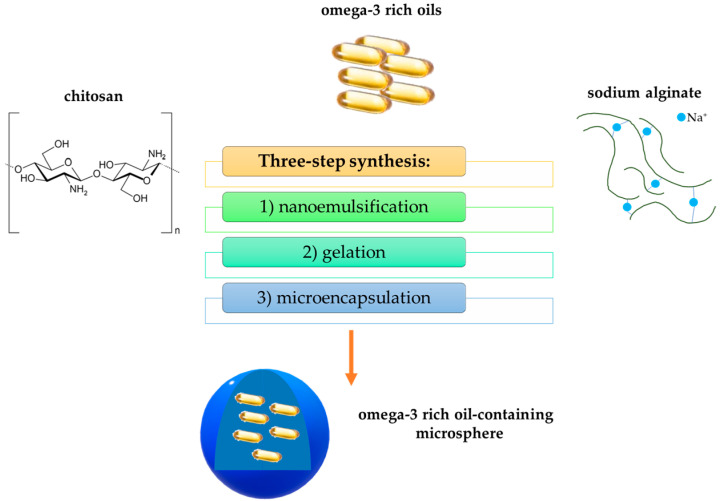
Scheme of the preparation of sodium alginate/chitosan-based microspheres.

In turn, Hamed et al. [103] analyzed nanocomposite microspheres based on alginate and chitosan in terms of their potential application in the delivery of omega-3-rich oils (like fish oils or flaxseed). These substances have numerous benefits and desirable properties, including antioxidant, anti-thrombotic, anti-inflammatory, and antimicrobial properties. However, they tend to oxidize. Thus, they should be protected against oxygen. This is the reason why their incorporation into adequate carriers is essential. Below, in Figure 7, the scheme of the preparation of microspheres is presented.

In this research, it was demonstrated that the developed nanocomposite microspheres turned out to be effective carriers for the mentioned active substances. A combination of alginate and chitosan has been also applied in the preparation of active substance carriers by Yu et al. [104]. They prepared nanocomposites as layered double hydroxide nanoparticles coated with both polysaccharides. Such designed systems were subsequently investigated as oral vaccine carriers. Based on the experiments performed, it was concluded that the developed coating may effectively protect oral vaccines against acidic/enzymatic degradation that may take place in the stomach. Chitosan/alginate-based nanocomposites containing additional cloister 30B were also investigated by Malesu et al. These studies also confirmed the effectiveness of carriers consisting of alginate and chitosan [105].

Alginate is also widely considered for developing curcumin carriers. Curcumin belongs to the group of natural polyphenols and shows numerous beneficial health properties, including antioxidant and anti-inflammatory properties [106]. It has been demonstrated that the encapsulation of curcumin increases its solvation in water [107]. For example, studies aimed at developing alginate-based nanocomposite carriers of curcumin were performed by Chegeni et al. [108]. In their research, they developed nanocomposites using calcium alginate and single-walled carbon nanotubes wherein the materials were additionally modified with glucose. The synthesis was carried out via the ultrasonication method. It was reported that the curcumin was released from the carriers in both the acidic (pH = 4.5) and neutral (pH = 7.5) environments. Importantly, the antibacterial properties of the developed carriers were also confirmed. 

Examples of alginate-based nanocomposites tested as carriers of selected active substances are presented below in Table 2.

Alginates are also used to develop carriers for the controlled delivery of tuberculosis drugs (e.g., rifampicin, ethionamide, isoniazid, pyrazinamide, ethambutol, amikacin, and moxifloxacin) [116]. Importantly, these polysaccharides are becoming more and more popular in developing carriers of chemotherapeutics, so drugs are being used in anti-cancer therapy. For example, nanocomposites based on alginate in combination with montmorillonite have been proposed as carriers of irinotecan [117]. This drug is widely used in the treatment of several types of cancers (including colon and rectal, lung, or ovarian cancer). In this study, the first step involved incorporating irinotecan into montmorillonite and the reaction of formed carriers with alginate via the ionotropic gelation technique. Next, the materials obtained were subjected to the in vitro release study wherein simulated intestinal fluid (37 °C; pH = 7.4) was employed for this purpose. The research results confirmed the sustained release of irinotecan without any burst effects, which is extremely important in chemotherapeutics. Lei et al. [118] developed nanocomposites based on sodium alginate, chitosan, and graphene oxide obtained via the electrostatic self-assembly process. The materials obtained were subsequently incorporated with doxorubicin hydrochloride and verified regarding their release ability. It was clearly reported that the formulated carriers effectively released the cytostatic drug within the desired area, thus showing cytotoxicity towards cancer cells. In other work, nanocomposite beads based on sodium alginate and montmorillonite have been tested as carriers of carboplatin [119]. Here, the nanocomposite materials were obtained via the ionotropic gelation technique. Based on the experiments performed, the prepared carriers’ potential in delivering the mentioned chemotherapeutic was confirmed.

The development of alginate-based drug carriers offers promising prospects but also raises potential challenges that require intensive research. Alginates, although biocompatible and biodegradable, can be susceptible to physicochemical changes in the biological environment, which can affect carrier stability and drug-release control [120]. Controlling the solubility of alginates under different conditions is also an issue, which is crucial for drug delivery efficiency. Moreover, challenges related to their bioavailability and selectivity in delivering drugs to specific cells or tissues must be considered [121].

In the chemistry of obtaining and designing alginate-based drug carriers, it is crucial to fine-tune the chemical composition of alginates to their intended therapeutic targets. The chemical composition of alginates is important in the process of designing drug carriers, as it affects their physicochemical properties, stability, and ability to interact with the active substance and biological environment. Proper selection of the type of alginate and its physicochemical properties significantly impact carriers’ ability to effectively store and release drugs [122,123,124]. Controlling the gelation process of alginates during synthesis is important for obtaining the desired structure of the carriers. Thus, the knowledge concerning the interactions between the alginates, active agents, and biological environment is crucial for the effective design of alginate-based drug carriers.

### 3.2. Cellulose-Based Nanocomposite Carriers

Cellulose is an organic chemical compound from the polysaccharide group, built from linear glucose chains linked by β-glycosidic bonds. It is the main component of plant cell walls, providing structure and strength. Chemically, cellulose is a polymer of glucose in which the unit sugar subunits are synchronized into long chains [125,126]. Cellulose fibers can form strong hydrogen bonds, giving them exceptional mechanical strength [127,128]. Cellulose is the primary raw material for paper and textile fiber fabrication [129,130].

Due to its unique properties, cellulose is used in medicine to develop drug carriers, especially in nanocomposites. Its ability to form structures with a large surface area enables efficient drug storage and transport. In addition, cellulose is biocompatible, which minimizes immune reactions when used in humans. Its solubility in certain solvents allows for the controlled release of the active ingredient from the carrier, which is crucial in drug therapy. In addition, cellulose exhibits low toxicity [131,132,133].

For example, studies on cellulose-based nanocomposites designed for drug delivery have been performed by Prusty and Swain [134]. In this work, cellulose-grafted polyacrylamide-based nanocomposites incorporated with gold nanoparticles were investigated regarding their potential application as carriers of ciprofloxacin, i.e., an antibiotic drug. It was concluded that 96.6% of this drug was released from the formulated nanocomposite carriers in 5 h. In turn, Ji et al. [135] examined bacterial cellulose/sodium alginate-based nanocomposite hydrogels as carriers of proteins (bovine serum albumin (BSA) and lysozymes). Based on this research, it was reported that their developed nanocomposites demonstrated biocompatibility and drug-release ability. The release rate determined for lysozymes was higher than for BSA due to its weaker interactions with the nanocomposite matrix. In another paper, Rana et al. described studies on nanocomposites based on cellulose and polyaniline [136]. Non-cytotoxicity and electroactive properties characterized these nanocomposites. Hence, they constituted interesting materials for controlled drug delivery. It allowed for them to state that the drug-release rate from their formulated carriers depended strongly on the pH of the medium in which the release occurred. In the case of acidic conditions (pH = 2.2), 37% of active substances were released, whereas in the case of alkaline medium (pH = 11.0), 69%. Investigations by Shahzadi et al. [137] were, in turn, focused on determining the potential of copolymer hydrogels consisting of poly(acrylic acid) grafted onto cellulose nanocrystals and calcium oxide nanoparticles in drug delivery. Such formulated nanocomposites have been subsequently incorporated with doxorubicin (a chemotherapeutic drug). These studies demonstrated the effective loading capacity of the developed nanocomposites (99%), possibly due to the electrostatic interactions between the drug and the carrier. Importantly, over 57.9% of the active substance was released from the carrier in 24 h. Next, Abukhadra et al. developed nanocomposites based on cellulose and exfoliated bentonite as oxaliplatin carriers [138]. They reported that their prepared materials showed high drug-release rates in slightly acidic environments (pH = 5.5), i.e., 83.3%, and in neutral environments (pH = 7.4), i.e., 93.4%. Importantly, these values were achieved after 100 h of the research. Many investigations also show the high application potential of nanocellulose in developing effective drug carriers [139,140].

Recently, growing attention within the area of drug carriers is also directed towards a cellulose derivative, i.e., carboxymethylcellulose. Examples of carboxymethylcellulose-based nanocomposites investigated as carriers of selected active substances are indicated in Table 3.

Cellulose, while biocompatible and biodegradable, may exhibit a limited ability to release the active substances in a controlled manner. It can also be problematic to maintain the structural stability of cellulose-based carriers, especially under changing conditions of the biological environment, which affects their drug delivery efficiency. Additionally, there are challenges in controlling particle size, which is important for their bioavailability and drug delivery efficiency to target sites. Therefore, systematic analysis is essential for the full utilization of cellulose in the field of drug delivery [149,150]. 

In the chemistry of obtaining and designing cellulose-based drug carriers, it is crucial to precisely tailor the chemical composition of cellulose to the intended therapeutic targets. Adequate selection of the type of cellulose and its degree of polymerization has a significant impact on the ability of the carriers to efficiently store and release drugs [151,152,153]. Controlling the micro- and macro-molecular structures of cellulose during the preparation process is key to providing the carriers with the desired porosity and ability to interact with the active substance. 

**Figure 8:**
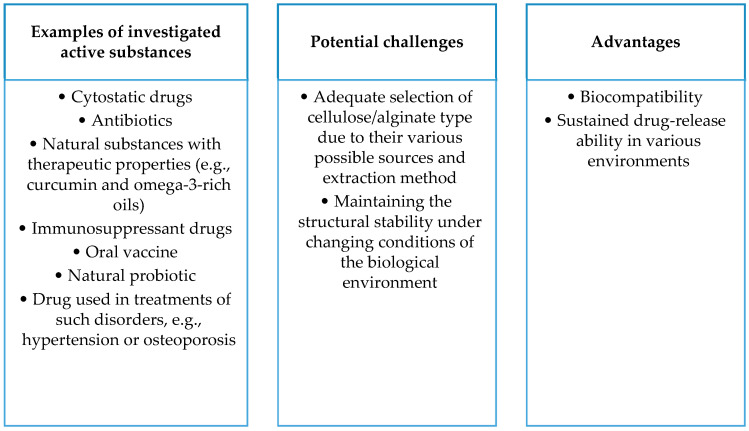
Summary of selected aspects of cellulose- and alginate-based drug carriers.

The figure below (Figure 8) summarizes the advantages of carriers based on the polysaccharides described in this section and the challenges accompanying the development of these carriers, as well as examples of drugs being tested.

Despite many potential challenges accompanying the development of carriers based on cellulose and alginates, these compounds show high application potential in terms of their application as carriers of various active substances. Other promising compounds within this area are proteins. The next section of this paper discusses two examples of these substances—gelatin and albumin.

## 4. Protein-Based Nanocomposite Carriers

### 4.1. Gelatin-Based Drug Carriers

Gelatin is a protein of animal origin, mainly extracted from the collagen of animal skin, bones, and cartilage. It is commonly used in the food industry as a thickener, gelling agent, and stabilizer in products such as jellies, puddings, and desserts. It is characterized by its ability to gel, which makes it useful in many culinary and technological processes [154,155]. This protein also finds application in the packaging industry, where coatings and films based on gelatin are becoming more and more popular due to their eco-friendly nature. Importantly, gelatin is prone to numerous modifications, including chemical, physical, irradiation, or enzymatic modifications, which additionally increase its application potential [156,157].

The ability of gelatin to form flexible gels in the presence of water and ease of structural modification enable the controlled release of active substances. Due to its ability to gel, gelatin provides an excellent matrix for incorporating drugs, allowing precise release rate adjustment. In addition, it is biocompatible and biodegradable, minimizing the risk of side effects. Moreover, gelatin can be used to produce microsphere drug carrier systems, ensuring stability and therapeutic efficacy [158,159,160].

**Figure 9:**
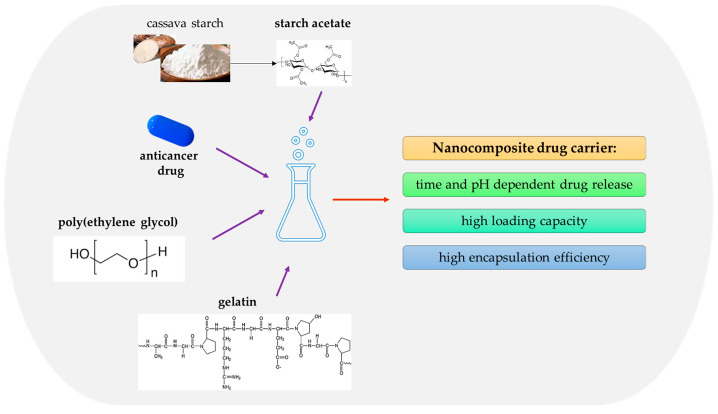
Components of a formulated nanocomposite and its properties.

Gelatin is widely considered for the preparation of cytostatic drug delivery systems. For example, Prabha and Raj [161] investigated nanocomposites based on gelatin, poly(ethylene glycol), and cassava starch acetate in terms of their application for the delivery of cisplatin. The mentioned carriers were obtained via the precipitation method. In Figure 9, the components of a formulated nanocomposite are presented, and its properties are significant in terms of its application as a drug carrier.

The drug-release ability of the formulated carriers was confirmed. The process was more effective in an acidic environment than an alkaline one due to the interactions between cassava starch acetate and the cytostatic drug. 

In turn, Najafabadi et al. [162] synthesized graphene oxide coated with gelatin and polyvinylpyrrolidone. Such obtained systems were subsequently incorporated with quercetin (showing anti-cancer activity) and dual water/oil/water nanoemulsions containing additionally bitter almond oil. As a result of their research, it was concluded that the formulated carriers exhibited high encapsulation efficiency (87.5%) and drug loading (45%). Moreover, their developed systems also showed high stability and drug-release ability, which was proven during their biological research. Cytotoxicity assay results demonstrated the death of over 53% of tested cancer cells treated with the materials obtained. Other researchers [163] received mesoporous silicate MCM-41-based nanocomposites additionally coated with gelatin and Pluronic^®^ F127 and incorporated them with doxorubicin. These conducted studies confirmed the release capability of nanocomposites wherein the process most effectively occurred in the environment with pH = 5.4 and at 42 °C. Importantly, the formulated carriers showed cytotoxicity towards cancer cells. In turn, in vivo tests performed using mice confirmed the anti-cancer activity of the developed materials, reflected in liver cancer growth suppression. Other studies [164] concerned the development of a delivery system for curcumin, an anti-cancer active substance of natural origin that is effective in the treatment of various types of cancer. Here, hydrogel nanocomposites of gelatin, chitosan, and carbon quantum dots were developed. The materials were synthesized via physical cross-linking and subjected to numerous studies, including, among others, the verification of both drug loading (DLE) and encapsulation (EE) efficiency. It was demonstrated that these hydrogel nanocomposites exhibited drug-release capability. The value of DLE was 87.5%, while that for EE was 46.75%. Furthermore, the formulated carriers showed cytotoxicity towards cancer cell lines.

In the next work [165], studies on magneto-responsive nanocomposite hydrogels based on gelatin and magnetic ion liquid surfactants were carried out. This approach assumed investigating such an obtained material as the carrier of both 5-fluorouracil (an anti-cancer drug) and ornidazole (an antibiotic). The experiments reported the biocompatibility of the prepared materials and high encapsulation efficiency. These formulated carriers’ properties combined with the simultaneous possibility of their manipulation via the external magnetic field make them very interesting within the area of controlled cytostatic delivery systems. Moya-Lopez et al. [166] presented studies on gelatin-based nanocomposites containing polylactide nanoparticles incorporated with doxorubicin and dasatinib, two drugs showing anti-cancer activity. The application potential of the formulated systems as drug carriers was confirmed. Moreover, it was stated that gelatin increased the biocompatibility of polymer nanoparticles and promoted cellular growth, thus enhancing the developed materials with additional therapeutic properties.

Many investigations have been conducted to develop gelatin-based nanocomposites as carriers of drugs showing antibacterial, anti-inflammatory, analgesic, or antipyretic activity. In Table 4, a compilation of such nanocomposites is presented.

Some works using gelatin have also been conducted to develop drug carriers for neurological or cardiological diseases. For example, Rahmani et al. [176] formulated nanocomposite hydrogels whose network consisted of poly(vinyl alcohol), gum arabic aldehyde, gelatin, graphene oxide, and boric acid and investigated in terms of rivastigmine delivery. This drug is widely used in the treatment of Alzheimer’s disease. As a result of the performed investigations, it was concluded that the formulated systems showed drug-release capability wherein this process took place in the environment with pH = 7.4. In another work, Jaberifard et al. [177] obtained gelatin-based microspheres incorporated with carvedilol-loaded halloysite nanotubes. Carvedilol is widely used in the treatment of coronary artery diseases, hypertension, and congestive treat heart failure. Based on the experiments performed, it was demonstrated that the formulated carriers showed drug-release ability in the environment with pH = 1.2 and may be considered oral delivery systems. Mathew and Arumainathan [178] described the synthesis and characterization of gelatin/chitosan-based nanocomposites investigated as dopamine carriers. Dopamine is applied, among others, in the treatment of Parkinson’s disease. It was reported that the developed materials showed high application potential for sustained delivery of the mentioned active substance. Importantly, these formulated nanocomposites also demonstrated antibacterial activity.

The development of gelatin-based drug carriers is drawing a promising branch of nanotechnology but faces potential challenges that require in-depth research. Although gelatin exhibits high biocompatibility, its stability under physicochemical conditions should be widely investigated [179,180]. Monitoring solubility in diverse biological environments is a key challenge, affecting structural stability and controlled drug release. Furthermore, maintaining the shelf life of gelatin carriers during storage and controlling particle size, which affects their bioavailability, is an important issue [181,182]. Further research is needed to understand the mechanisms of degradation and release of the active substance during gelatin degradation.

When developing and constructing gelatin-based drug nanocarriers, a key aspect is to precisely tailor the chemical composition of gelatin to the intended therapeutic targets. Appropriate selection of the gelatin source, thermal processes, and nanotechnology techniques affects the ability of the carriers to efficiently carry and control drug release. Thermal processes and nanotechnology techniques play a key role in obtaining and designing gelatin-based drug nanocarriers. Thermal processes involve the controlled heating of gelatin, which allows it to be transformed into a more flexible and stable form, perfectly suited for nanocarriers. Nanotechnology techniques, such as emulsification, allow for the precise control of the size and morphology of nanocarriers, which is crucial for their ability to carry and release drugs. The combination of these processes makes it possible to construct gelatin nanocarriers with optimized properties, as well as enabling controlled drug delivery in the body [183,184].

### 4.2. Albumin-Based Drug Carriers

Albumins are a family of proteins found in the bodies of many organisms, including humans. Chemically, they are globular proteins comprising over 500 amino acids. There are many types of albumin, including ovalbumin, bovine serum albumin, and human serum albumin [185,186]. In humans, albumin, particularly human serum albumin, plays a key role in maintaining oncotic pressure and transporting substances such as hormones, fatty acids, and drugs. Due to its unique properties, albumin can find wide application in medicine, especially in developing drug carriers. Its ability to specifically bind and transport various molecules allows for controlled drug release, which is crucial in therapy [187,188,189]. Hence, the application potential of albumin in the controlled delivery of various molecules has been investigated since the mid-1990s. Many studies have verified this protein as a substance that can deliver drugs for malignant or inflammatory tissues. Importantly, albumin shows biodegradability and biocompatibility, significantly reducing the risk of immune reactions, and is meaningful considering its potential use as a drug carrier [190,191].

In many studies, the affinity of albumin for biodistribution within cancer cells and the uptake of this protein by these cells have been proven [192,193]. Therefore, many studies have been performed to develop albumin-containing carriers of chemotherapeutic drugs to provide drug accumulation exactly within the affected site. In Table 5, a compilation of investigated chemotherapeutic carriers is presented.

Numerous investigations are also being conducted on applying albumin-containing carriers in delivering active substances that are not only intended for cancer treatment. For example, Jalali et al. [202] investigated nanocomposites based on bovine serum albumin and oxidized Arabic gum. These materials were subsequently loaded with piperine, so the drug showed antibacterial, analgesic, and antifungal activity. Based on their research, it was concluded that the formulated carriers showed high loading efficiency and sustained drug-release capability. Importantly, the materials obtained demonstrated cytotoxicity towards the tested cell lines. In turn, Jing et al. [203] carried out studies on albumin-based nanocomposites incorporated with N5,N6-Bis(2-fluorophenyl)[1,2,5]oxadiazolo[3,4-b]pyrazine-5,6-diamine (BAM15), an active substance that may prevent obesity and its potential symptoms. The experiments performed demonstrated the excellent biocompatibility of prepared carriers and their liver targeting capability, possibly due to the presence of albumin within their structure. Moreover, strong anti-obesity activity was observed without simultaneously affecting food intake and altering body temperature.

It is important to develop albumin-containing nanoscale drug delivery systems to control particle size and aggregation phenomena [204]. The mentioned parameters may affect the bioavailability and therapeutic efficacy of albumin. Therefore, further investigations on this protein are essential in the context of using albumin as a drug carrier. In the process of obtaining and designing albumin-based drug nanocarriers, it is crucial to tailor the chemical composition of albumin precisely to the intended therapeutic targets. The optimal choice of albumin source and chemical modification method affects the ability of the carriers to efficiently carry and control drug release. Investigating and monitoring the size and morphology of the nanocarriers during the preparation process are key to obtaining carriers with optimized drug delivery properties [205,206].

**Figure 10:**
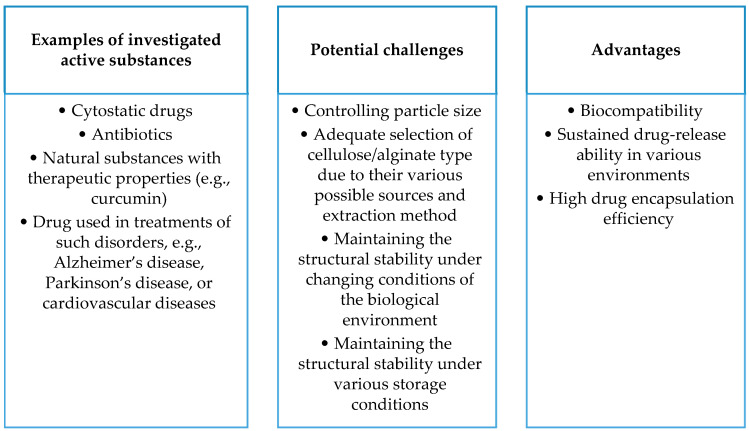
Summary of selected aspects of gelatin- and albumin-based drug carriers.

The figure below (Figure 10) condenses the advantages of carriers based on albumin and gelatin, the challenges accompanying the development of the carriers, and examples of drugs being investigated.

Some challenges may be discussed considering studies on the development of drug carriers based on albumin and gelatin. Nonetheless, many advantages of these proteins undoubtedly speak for their potential within this field.

## 5. Summary

### 5.1. Conclusions

- In today’s world, the development of modern therapies and active substance delivery strategies requires innovative approaches. Traditional drug delivery methods, such as oral, intravenous, transdermal, or muscle delivery of the active ingredient, have significant limitations, including degradation in the gastrointestinal tract. Lack of selectivity and susceptibility to side effects underscore the need for novel solutions. Bionanocomposites are advanced drug carriers, enabling the precise and targeted delivery of active substances, which could revolutionarily improve the efficacy of therapies, especially in cancer treatment.
- Liposomes represent a promising drug carrier, enabling the encapsulation of both hydrophilic and hydrophobic substances. Their ability to precisely deliver active substances opens new perspectives in anti-cancer therapy, accelerating wound healing or delivering drugs to the eye. In addition, liposomes allow for the controlled release of active substances, which can increase drug stability and reduce side effects.
- Lipid nanoemulsions offer stable solutions for improving the solubility of lipophilic substances. Their ability to efficiently transport active substances, especially in terms of improving bioavailability, makes them attractive drug carriers. In addition, nanoemulsions can be customized, allowing for them to be used in a variety of therapeutic areas, such as the delivery of anti-cancer drugs or the treatment of gastrointestinal diseases.
- Carriers such as solid lipid nanoparticles (SLNs) and nanostructured lipid carriers (NLCs) represent advanced strategies in drug delivery. Their stability, controlled release of active substances, and ability to improve bioavailability make them promising tools in the field of drug therapy. The use of SLNs, which are constructed from lipids in solid form, and NLCs, which combine liquids and solid oils, presents new possibilities in the efficient transport of drugs, especially in the context of anti-cancer therapy or the delivery of lipophilic substances.
- Sodium alginate, being biodegradable, biocompatible, and readily available, holds promise as a material for drug delivery systems. Alginate-based nanocomposites, such as composites with hydroxyapatite and ciprofloxacin, exhibit controlled drug release, enhancing therapeutic efficacy. Alginate-based nanocomposites also show promise as carriers for curcumin, increasing its solubility and demonstrating potential antibacterial properties. Alginate is applied in delivering drugs against tuberculosis and in anti-cancer therapy, indicating its significant potential in medicine.
- Cellulose, being biocompatible and suitable as a drug carrier, demonstrates potential in delivering antibiotics, especially in nanocomposites with gold nanoparticles. Copolymer hydrogels with cellulose nanocrystals effectively transport doxorubicin, showing controlled drug release. Nanocellulose gains recognition as an effective drug carrier, indicating a promising path for future research.
- Gelatin, with its ability to form matrices for active substances, is emerging as a promising material in the field of drug delivery. Its biocompatibility, biodegradability, and ability to form drug carrier systems, such as microspheres, may increase drug stability and improve their therapeutic efficacy. Moreover, this protein is being intensively studied as a component of nanocomposite carriers of anti-cancer drugs. Incorporating active compounds, such as cisplatin, quercetin, or doxorubicin, into gelatin-based nanocarriers shows promise regarding drug-release efficiency and their cytotoxic effects against cancer cells.
- Albumin has potential for controlled drug release due to its ability to specifically bind and transport various molecules. In addition, albumin exhibits biodegradability and biocompatibility, significantly reducing the risk of immune reactions, which is important for its potential use as a drug carrier. Many studies have confirmed the potential of albumin to deliver drugs to tumor tissues. The ability of albumin to be biodistributed across cancer cells and the ability of these cells to internalize the protein have been demonstrated. This finding indicates the potential for using albumin in targeted drug delivery to cancer cells. Research on albumin-containing drug carriers is not just focused on cancer therapy. Numerous studies show the potential of albumin in the delivery of antibacterial, analgesic, and antifungal drugs, as well as active substances to prevent obesity.

### 5.2. Perspectives and Future Challenges

- Studies on proteins, polysaccharides, and lipid-containing nanosystems (including liposomes, lipid nanoemulsions, solid lipid nanoparticles, and nanostructured lipid carriers) show promising results in terms of therapeutic efficacy and improvement in the stability and bioavailability of active substances. Future research may focus on further refining nanocomposite technologies, increasing their specificity and effectiveness in drug delivery across various medical, pharmacological, and cosmetic applications.
- The development of drug carriers based on proteins, polysaccharides, and lipids promises to improve therapeutic efficacy but brings with it complex issues. Liposomes can disintegrate and react with digestive enzymes, requiring in-depth studies in the context of stability, release, and interaction with the immune system. Analogous issues apply to nanoemulsions. In the case of solid lipid nanoparticles (SLNs) and nanostructured lipid carriers (NLCs), difficulties related to stability, particle size control, and biological interactions are significant. Despite the high biocompatibility of gelatin, further research is needed to maintain it under physicochemical conditions, and solubility regulation is a key challenge. Polysaccharides, such as alginates, face difficulties in terms of physicochemical stability in biological environments, affecting the persistence and control of drug release. Cellulose, despite its biocompatibility, requires research on maintaining stability under physicochemical conditions, especially control of solubility in different biological environments. Issues related to maintaining the stability of cellulose carriers during storage, controlling particle size, and affecting bioavailability are areas for further research.

## Figures and Tables

**Table 1:** Drug combinations with liposomes for use in therapeutic therapies.

Structure	Nanocomposite Matrix	Drug/Active Substance	Application	Ref.
Liposome	Soy lecithin, cetyltrimethylammonium chloride phosphate buffer, ZnFe_2_O_4_, and hyaluronic acid	Imatinib	drug delivery (anti-cancer therapy)	[50]
Liposome	Poly(vinyl alcohol) and chitosan	Taxifolin	Accelerating wound healing in diabetic patients	[51]
Liposome	Hydroxypropyl-β-cyclodextrin	Dexamethasone	Topical drug delivery system for the posterior segment of the eye	[52]
Liposome	Gelatin and methacrylate	Chemokinin SDF-1α	Stimulation of cell migration	[53]
Liposome	Ag/Au	Doxorubicin	Drug delivery (anti-cancer therapy)	[54]
Liposome	Fullerene and PEGylated iron oxide nanoparticles	Doxorubicin	Multi-mechanism cancer treatment based on radiofrequency-induced imaging and targeted drug delivery via an external magnetic field	[55]
Liposome	Folic acid and gold nanorods	Doxorubicin	Cancer treatment via both chemotherapy and photothermal therapy	[56]

**Table 2:** Examples of investigated alginate-based nanocomposite carriers presented with tested active substances and their properties.

Active Agent	Active Substance Properties	Nanocomposite Structure	Ref.
Prednisolone	Immunosuppressant drug used to treat some inflammatory diseases and some types of cancer	Poly(vinyl pyrrolidone)/sodium alginate copolymer incorporated with silver nanoparticles	[109]
*Lactobacillus rhamnosus* GG	Natural probiotic positively altering the gut microbiome composition	Exfoliated bentonite/alginate nanocomposite hydrogels	[110]
Amlodipine besylate	Calcium channel blocker applied in angina and hypertension	Nanocomposite matrix based on alginate, chitosan, and graphene oxide incorporated with inclusion complexes of amlodipine besylate and β-Cyclodextrin	[111]
Edaravone	Free radical scavenger approved for acute cerebral infarction treatment	Nanocomposite hydrogels based on alginate and positively charged Eudragit nanoparticles incorporated with the drug	[112]
Propranolol	Drug applied for cardiac treatment; shows anti-anxiety and anti-migraine effects	Sodium alginate/pectin/tannic acid—silver nanoparticle-based nanocomposite prepared via microwave irradiation	[113]
Alendronate sodium	Drug applied for osteoporosis treatment	Sodium alginate cross-linked montmorillonite nanocomposite beads	[114]
Chlorhexidine digluconate	Drug with antibacterial activity applied in dentinal tubules infections	Alginate/nanocellulose-based nanocomposites	[115]
Tofacitinib	Drug applied for autoimmune disease treatment	Alginate-based beads containing drug-incorporated nanoemulsions	[69]

**Table 3:** Examples of carboxymethylcellulose-based nanocomposite carriers compiled with active substances and their properties.

Active Agent	Active Substance Properties	Nanocomposite Structure	Ref.
Doxorubicin	Anti-cancer drug	Carboxymethyl cellulose/ZnO/starch-based nanocomposite hydrogel beads	[141]
Doxorubicin	Anti-cancer drug	Nanocomposites based on carbon dots conjugated carboxymethyl cellulose and hydroxyapatite	[142]
Doxorubicin	Anti-cancer drug	Nanocomposite hydrogel based on graphene quantum dot crosslinked carboxymethyl cellulose	[143]
Doxorubicin	Anti-cancer drug	Nanocomposite hydrogel beads based on carboxymethyl cellulose/graphene oxide	[144]
Artesunate	Anti-malarial drug also showing anti-cancer efficacy	Nanocomposites based on polyhydroxybutyrate and functionalized carboxymethylcellulose and additionally containing zinc oxide and Fe_3_O_4_ magnetic nanoparticles	[145]
5-fluorouracil (5-FU)	Anti-cancer drug	Nanocomposite hydrogel beads based on carboxymethylcellulose and Arabic gum	[146]
Tetracycline	Antibiotic	Nanocomposite based on carboxymethyl cellulose containing Zn-melamine and Cu-melamine framework	[147]
Diclofenac sodium	Nonsteroidal anti-inflammatory drug	Nanocomposite based on poly(methacrylic acid) crosslinked with carboxymethyl cellulose and incorporated with in situ-formed silver nanoparticles	[148]

**Table 4:** Examples of gelatin-based nanocomposite carriers with tested drug/active substances and their properties.

Active Substance	Active Substance Properties	Nanocomposite Structure	Ref.
Acetaminophen (paracetamol)	Analgesic and antipyretic activity	Gelatin-based nanocomposite hydrogel incorporated with drug-loaded poly(N–isopropylacrylamide) nanoparticles	[167]
Cefadroxil	Antibacterial activity	Gelatin-based nanocomposites incorporated with carbon dots	[168]
Cephalexin	Antibacterial activity	chemically crosslinked gelatin-based hydrogel nanocomposites incorporated with CuO nanoparticles	[169]
Ciprofloxacin	Antibacterial activity	Gelatin, starch, and itaconic acid-based hydrogel nanocomposites containing ZnO and cellulose nanofibers	[170]
Ciprofloxacin	Antibacterial activity	Gelatin-grafted polyacrylamide nanocomposite hydrogels containing silver nanoparticles and carbon dots	[171]
Ibuprofen	Analgesic, anti-inflammatory, and antipyretic activity	Gelatin/carboxymethyl chitosan/graphene oxide-based nanocomposite hydrogel	[172]
Flurbiprofen	Analgesic, anti-inflammatory, and antipyretic activity	Dual crosslinked gelatin/gellan gum-based nanocomposite hydrogel incorporated with cerium oxide nanoparticles	[173]
*Camellia sinensis*	Herbal drug showing antibacterial activity	Nanocomposite hydrogel based on methacrylic anhydride, modified gelatin, and an iron-based metal–organic framework	[174]
Dexamethasone	Anti-inflammatory, analgesic, anti-allergic, and immunosuppressive activity	Gelatin methacryloyl/nanosilicate-based nanocomposite hydrogels	[175]

**Table 5:** Examples of albumin-containing nanocomposite carriers designed for anti-cancer therapy.

Drug/Active Substance	Application	Nanocomposite Structure	Ref.
Doxorubicin	Anti-cancer therapy	Folic acid-grafted bovine serum albumin/graphene oxide-based nanocomposite	[194]
Saponin	Colorectal cancer treatment	Montmorillonite loaded with saponine/human serum albumin-based nanocomposite	[195]
Doxorubicin	Anti-cancer therapy	Bactrian camel serum albumin-based nanocomposite	[196]
Etoposide	Lung cancer treatment	Boronic acid-modified albumin-based nanocomposites	[197]
Doxorubicin, gambogic acid	Liver cancer treatment	Albumin-based nanocomposites	[198]
5-fluorouracil	Liver cancer treatment	Nanocomposites consisting of folic acid, bovine serum albumin, layered double hydroxide, and quantum-sized Fe_3_O_4_	[199]
Doxorubicin	Lung cancer treatment	Bactrian camel serum albumin-based nanocomposites incorporated with glutathione-responsive curcumin	[200]
5-fluorouracil, curcumin	Colorectal cancer treatment	Nanocomposites based on graphene oxide and folic acid-functionalized albumin	[201]

## Data Availability

Not applicable.

## References

[1] Kandula S., Singh P.K., Kaur G.A., Tiwari A. (2023). Trends in smart drug delivery systems for targeting cancer cells. Mater. Sci. Eng. B.

[2] Srivastav A.K., Karpathak S., Rai M.K., Kumar D., Misra D.P., Agarwal V. (2023). Lipid based drug delivery systems for oral, transdermal and parenteral delivery: Recent strategies for targeted delivery consistent with different clinical application. J. Drug Deliv. Sci. Technol..

[3] Adepu S., Ramakrishna S. (2021). Controlled Drug Delivery Systems: Current Status and Future Directions. Molecules.

[4] Sultana A., Zare M., Thomas V., Sampath Kumar T.S., Ramakrishna S. (2022). Nano-based drug delivery systems: Conventional drug delivery routes, recent developments and future prospects. Med. Drug Discov..

[5] Hua S. (2020). Advances in oral drug delivery for regional targeting in the gastrointestinal tract-influence of physiological, pathophysiological and pharmaceutical factors. Front. Pharmacol..

[6] Ezike T.C., Okpala U.S., Onoja U.L., Nwike C.P., Ezeako E.C., Okpara O.J., Okoroafor C.C., Eze S.C., Kalu O.L., Odoh E.C. (2023). Advances in drug delivery systems, challenges and future directions. Heliyon.

[7] Tewabe A., Abate A., Tamrie M., Seyfu A., Abdela Siraj E. (2021). Targeted Drug Delivery—From Magic Bullet to Nanomedicine: Principles, Challenges, and Future Perspectives. J. Multidiscip. Healthc..

[8] Veselov V.V., Nosyrev A.E., Jicsinszky L., Alyautdin R.N., Cravotto G. (2022). Targeted Delivery Methods for Anticancer Drugs. Cancers.

[9] Yang L., Yang Y., Chen Y., Xu Y., Peng J. (2022). Cell-based drug delivery systems and their in vivo fate. Adv. Drug Deliv. Rev..

[10] Zhao Z., Ukidve A., Kim J., Mitragotri S. (2020). Targeting strategies for tissue-specific drug delivery. Cell.

[11] Wu J. (2021). The enhanced permeability and retention (EPR) effect: The significance of the concept and methods to enhance its application. J. Pers. Med..

[12] Sharma G., Sharma A.R., Lee S.S., Bhattacharya M., Nam J.S., Chakraborty C. (2019). Advances in nanocarriers enabled brain targeted drug delivery across blood brain barrier. Int. J. Pharm..

[13] Chen L., Hong W., Ren W., Xu T., Qian Z., He Z. (2021). Recent progress in targeted delivery vectors based on biomimetic nanoparticles. Signal Transduct. Target. Ther..

[14] Wen P., Ke W., Dirisala A., Toh K., Tanaka M., Li J. (2023). Stealth and pseudo-stealth nanocarriers. Adv. Drug Deliv. Rev..

[15] Manzari M.T., Shamay Y., Kiguchi H., Rosen N., Scaltriti M., Heller D.A. (2021). Targeted drug delivery strategies for precision medicines. Nat. Rev. Mater..

[16] Finbloom J.A., Sousa F., Stevens M.M., Desai T.A. (2020). Engineering the drug carrier biointerface to overcome biological barriers to drug delivery. Adv. Drug Deliv. Rev..

[17] Di J., Gao X., Du Y., Zhang H., Gao J., Zheng A. (2021). Size, shape, charge and “stealthy” surface: Carrier properties affect the drug circulation time in vivo. Asian J. Pharm. Sci..

[18] Trucillo P. (2021). Drug carriers: Classification, administration, release profiles, and industrial approach. Processes.

[19] Yoo J., Park C., Yi G., Lee D., Koo H. (2019). Active targeting strategies using biological ligands for nanoparticle drug delivery systems. Cancers.

[20] Nelemans L.C., Gurevich L. (2020). Drug delivery with polymeric nanocarriers—Cellular uptake mechanisms. Materials.

[21] Li J., Wang Q., Xia G., Adilijiang N., Li Y., Hou Z., Fan Z., Li J. (2023). Recent Advances in Targeted Drug Delivery Strategy for Enhancing Oncotherapy. Pharmaceutics.

[22] Bhatia S., Bhatia S. (2016). Natural polymers vs. synthetic polymer. Natural Polymer Drug Delivery Systems: Nanoparticles, Plants, and Algae.

[23] Mogoşanu G.D., Grumezescu A.M., Bejenaru L.E., Bejenaru C., Grumezescu A.M. (2016). Chapter 8—Natural and synthetic polymers for drug delivery and targeting. Nanobiomaterials in Drug Delivery. Applications of Nanobiomaterials.

[24] Tong X., Pan W., Su T., Zhang M., Dong W., Qi X. (2020). Recent advances in natural polymer-based drug delivery systems. React. Funct. Polym..

[25] George A., Shah P.A., Shrivastav P.S. (2019). Natural biodegradable polymers based nano-formulations for drug delivery: A review. Int. J. Pharm..

[26] Raveendran S., Rochani A.K., Maekawa T., Kumar D.S. (2017). Smart carriers and nanohealers: A nanomedical insight on natural polymers. Materials.

[27] Liu D., Yang F., Xiong F., Gu N. (2016). The smart drug delivery system and its clinical potential. Theranostics.

[28] Khan I., Khan M., Umar M.N., Oh D.H. (2015). Nanobiotechnology and its applications in drug delivery system: A review. IET Nanobiotechnol..

[29] Xiao R., Zhou G., Wen Y., Ye J., Li X., Wang X. (2023). Recent advances on stimuli-responsive biopolymer-based nanocomposites for drug delivery. Compos. B Eng..

[30] Omanovic-Miklicanin E., Badnjević A., Kazlagić A., Hajlovac M. (2020). Nanocomposites: A brief review. Health Technol..

[31] Chen J., Ashames A., Buabeid M.A., Fahelelbom K.M., Ijaz M., Murtaza G. (2020). Nanocomposites drug delivery systems for the healing of bone fractures. Int. J. Pharm..

[32] Jayakumar A., Mathew S., Radoor S., Kim J.T., Rhim J., Siengchin S. (2023). Recent advances in two-dimensional nanomaterials: Properties, antimicrobial, and drug delivery application of nanocomposites. Mater. Today Chem..

[33] Ali A., Ahmed S. (2018). A review on chitosan and its nanocomposites in drug delivery. Int. J. Biol. Macromol..

[34] Inamuddin A., Mohammad A. (2018). Applications of Nanocomposite Materials in Drug Delivery Sawston.

[35] Harugade A., Sherje A.P., Pethe A. (2023). Chitosan: A review on properties, biological activities and recent progress in biomedical applications. React. Funct. Polym..

[36] Jeevanandam J., Rodrigues J., Pan S., Danquah M.K., Sharma B., Thomas S., Bajpai P.K., Ghosal K., Shekhar S. (2024). Chapter 8—Cellulose-based bionanocomposites: Synthesis, properties, and applications. Advances in Bionanocomposites. Materials, Applications, and Life Cycle. Micro and Nano Technologies.

[37] Kang J.H., Ko Y.T. (2015). Lipid-coated gold nanocomposites for enhanced cancer therapy. Int. J. Nanomed..

[38] Puglia C., Lauro M.R., Tirendi G.G., Fassari G.E., Carbone C., Bonina F., Puglisi G. (2017). Modern drug delivery strategies applied to natural active compounds. Expert Opin. Drug Deliv..

[39] Kumari A., Singla R., Guliani A., Yadav S.K. (2014). Nanoencapsulation for drug delivery. EXCLI J..

[40] Amiri M., Khazaeli P., Salehabadi A., Salavati-Niasari M. (2021). Hydrogel beads-based nanocomposites in novel drug delivery platforms: Recent trends and developments. Adv. Colloid Interface Sci..

[41] Mumtaz S., Khattak S., Rehman F.U., Muhammad P., Hanif S., Das S., Thomas S., Das P.P. (2023). Chapter 13—Bionanocomposites as a new platform for drug delivery systems. Woodhead Publishing Series in Biomaterials. Novel Platforms for Drug Delivery Applications.

[42] Çalış S., Atar K.Ö., Arslan F.B., Eroğlu H., Çapan Y., Mohapatra S.S., Ranjan S., Dasgupta N., Mishra R.K., Thomas S. (2019). Chapter 4—Nanopharmaceuticals as Drug-Delivery Systems: For, Against, and Current Applications. Micro and Nano Technologies. Nanocarriers for Drug Delivery.

[43] Seo Y., Lim H., Park H., Yu J., An J., Yoo H.Y., Lee T. (2023). Recent Progress of Lipid Nanoparticles-Based Lipophilic Drug Delivery: Focus on Surface Modifications. Pharmaceutics.

[44] Manning S.R. (2022). Microalgal lipids: Biochemistry and biotechnology. Curr. Opin. Biotechnol..

[45] Guo R., Chen Y., Borgard H., Jijiwa M., Nasu M., He M., Deng Y. (2020). The Function and Mechanism of Lipid Molecules and Their Roles in The Diagnosis and Prognosis of Breast Cancer. Molecules.

[46] Kerr B.J., Kellner T.A., Shurson G.C. (2015). Characteristics of lipids and their feeding value in swine diets. J. Anim. Sci. Biotechnol..

[47] Angellotti G., Presentato A., Murgia D., Di Prima G., D’Agostino F., Scarpaci A.G., D’Oca M.C., Alduina R., Campisi G., De Caro V. (2021). Lipid Nanocarriers-Loaded Nanocomposite as a Suitable Platform to Release Antibacterial and Antioxidant Agents for Immediate Dental Implant Placement Restorative Treatment. Pharmaceutics.

[48] Bozzuto G., Molinari A. (2015). Liposomes as nanomedical devices. Int. J. Nanomed..

[49] Nakhaei P., Margiana R., Bokov D.O., Abdelbasset W.K., Jadidi Kouhbanani M.A., Varma R.S., Marofi F., Jarahian M., Beheshtkhoo N. (2021). Liposomes: Structure, Biomedical Applications, and Stability Parameters with Emphasis on Cholesterol. Front. Bioeng. Biotechnol..

[50] Amiri M., Gholami T., Amiri O., Pardakhti A., Ahmadi M., Akbari A., Salavati-Niasari M. (2020). The magnetic inorganic-organic nanocomposite based on ZnFe_2_O_4_-Imatinib-liposome for biomedical applications, in vivo and in vitro study. J. Alloys Compd..

[51] Ding Q., Ding C., Liu X., Zheng Y., Zhao Y., Zhang S., Liu W. (2023). Preparation of nanocomposite membranes loaded with taxifolin liposome and its mechanism of wound healing in diabetic mice. Int. J. Biol. Macromol..

[52] Lu J., Zhu X., Zhang M., Jiang X., Guo W., Jiang F., Cao F. (2023). In vitro and in vivo assessment of structural integrity for HPCD complex@ Liposome nanocomposites from ocular surface to the posterior segment of the eye. Carbohydr. Polym..

[53] Justine R.Y., Janssen M., Liang B.J., Huang H.C., Fisher J.P. (2020). A liposome/gelatin methacrylate nanocomposite hydrogel system for delivery of stromal cell-derived factor-1α and stimulation of cell migration. Acta Biomater..

[54] Zhao Y., Zhao J., Shan G., Yan D., Chen Y., Liu Y. (2017). SERS-active liposome@ Ag/Au nanocomposite for NIR light-driven drug release. Colloids Surf. B Biointerfaces.

[55] Zhang N., Wu Y., Xu W., Li Z., Wang L. (2022). Synergic fabrication of multifunctional liposomes nanocomposites for improved radiofrequency ablation combination for liver metastasis cancer therapy. Drug Deliv..

[56] Nguyen V.D., Min H., Kim C., Han J., Park J., Choi E. (2019). Folate receptor-targeted liposomal nanocomplex for effective synergistic photothermal-chemotherapy of breast cancer in vivo. Colloids Surf. B Biointerfaces.

[57] Yu J.Y., Chuesiang P., Shin G.H., Park H.J. (2021). Post-processing techniques for the improvement of liposome stability. Pharmaceutics.

[58] Guimarães D., Cavaco-Paulo A., Nogueira E. (2021). Design of liposomes as drug delivery system for therapeutic applications. Int. J. Pharm..

[59] Šturm L., Poklar Ulrih N. (2021). Basic Methods for Preparation of Liposomes and Studying Their Interactions with Different Compounds, with the Emphasis on Polyphenols. Int. J. Mol. Sci..

[60] Sawaftah N.A., Paul V., Awad N., Husseini G.A. (2021). Modeling of Anti-Cancer Drug Release Kinetics from Liposomes and Micelles: A Review. IEEE Trans..

[61] Zahednezhad F., Saadat M., Valizadeh H., Zakeri-Milani P., Baradaran B. (2019). Liposome and immune system interplay: Challenges and potentials. J. Control. Release.

[62] Inglut C.T., Sorrin A.J., Kuruppu T., Vig S., Cicalo J., Ahmad H., Huang H.C. (2020). Immunological and toxicological considerations for the design of liposomes. Nanomaterials.

[63] Li J., Wang X., Zhang T., Wang C., Huang Z., Luo X., Deng Y. (2015). A review on phospholipids and their main applications in drug delivery systems. Asian J. Pharm. Sci..

[64] Sakdiset P., Okada A., Todo H., Sugibayashi K. (2018). Selection of phospholipids to design liposome preparations with high skin penetration-enhancing effects. J. Drug Deliv. Sci. Technol..

[65] Nsairat H., Khater D., Sayed U., Odeh F., Al Bawab A., Alshaer W. (2022). Liposomes: Structure, composition, types, and clinical applications. Heliyon.

[66] Hörmann K., Zimmer A. (2016). Drug delivery and drug targeting with parenteral lipid nanoemulsions—A review. J. Control. Release.

[67] Parchekani J., Allahverdi A., Taghdir M., Naderi-Manesh H. (2022). Design and simulation of the liposomal model by using a coarse-grained molecular dynamics approach towards drug delivery goals. Sci. Rep..

[68] Sabjan K.B., Munawar S.M., Rajendiran D., Vinoji S.K., Kasinathan K. (2020). Nanoemulsion as Oral Drug Delivery—A Review. Curr. Drug Res. Rev..

[69] Andretto V., Taurino G., Guerriero G., Guérin H., Lainé E., Bianchi M.G., Lollo G. (2023). Nanoemulsions Embedded in Alginate Beads as Bioadhesive Nanocomposites for Intestinal Delivery of the Anti-Inflammatory Drug Tofacitinib. Biomacromolecules.

[70] Hinger D., Navarro F., Käch A., Thomann J.S., Mittler F., Couffin A.C., Maake C. (2016). Photoinduced effects of m-tetrahydroxyphenylchlorin loaded lipid nanoemulsions on multicellular tumor spheroids. J. Nanobiotechnol..

[71] Samadi A., Pourmadadi M., Yazdian F., Rashedi H., Navaei-Nigjeh M. (2021). Ameliorating quercetin constraints in cancer therapy with pH-responsive agarose-polyvinylpyrrolidone-hydroxyapatite nanocomposite encapsulated in double nanoemulsion. Int. J. Biol. Macromol..

[72] Ahmadi H., Pourmadadi M., Abdouss M., Rahdar A., Díez-Pascual A.M. (2023). Formulation of double nanoemulsions based on pH-sensitive poly acrylic acid/agarose/ZnO for quercetin controlled release. J. Mol. Liq..

[73] Shamsabadipour A., Pourmadadi M., Rashedi H., Yazdian F., Navaei-Nigjeh M. (2023). Nanoemulsion carriers of porous γ-alumina modified by polyvinylpyrrolidone and carboxymethyl cellulose for pH-sensitive delivery of 5-fluorouracil. Int. J. Biol. Macromol..

[74] Manzoor M., Sharma P., Murtaza M., Jaiswal A.K., Jaglan S. (2023). Fabrication, characterization, and interventions of protein, polysaccharide and lipid-based nanoemulsions in food and nutraceutical delivery applications: A review. Int. J. Biol. Macromol..

[75] Mushtaq A., Wani S.M., Malik A.R., Gull A., Ramniwas S., Nayik G.A., Ercisli S., Marc R.A., Bari A. (2023). Recent insights into Nanoemulsions: Their preparation, properties and applications. Food Chem. X.

[76] Choi S.J., McClements D.J. (2020). Nanoemulsions as delivery systems for lipophilic nutraceuticals: Strategies for improving their formulation, stability, functionality and bioavailability. Food Sci. Biotechnol..

[77] Singh Y., Meher J.G., Raval K., Khan F.A., Chaurasia M., Jain N.K., Chourasia M.K. (2017). Nanoemulsion: Concepts, development and applications in drug delivery. J. Control. Release.

[78] Marhamati M., Ranjbar G., Rezaie M. (2021). Effects of emulsifiers on the physicochemical stability of Oil-in-water Nanoemulsions: A critical review. J. Mol. Liq..

[79] Sen Gupta S., Ghosh M. (2015). Formulation development and process parameter optimization of lipid nanoemulsions using an alginate-protein stabilizer. J. Food Sci. Technol..

[80] Lingayat V.J., Zarekar N.S., Shendge R.S. (2017). Solid lipid nanoparticles: A review. Nanosci. Nanotechnol. Res..

[81] Samiun W.S., Ashari S.E., Salim N., Ahmad S. (2020). Optimization of Processing Parameters of Nanoemulsion Containing Aripiprazole Using Response Surface Methodology. Int. J. Nanomed..

[82] Naseri N., Valizadeh H., Zakeri-Milani P. (2015). Solid lipid nanoparticles and nanostructured lipid carriers: Structure, preparation and application. Adv. Pharm. Bull..

[83] Beloqui A., Solinís M.Á., Rodríguez-Gascón A., Almeida A.J., Préat V. (2016). Nanostructured lipid carriers: Promising drug delivery systems for future clinics. Nanomedicine.

[84] Vigani B., Valentino C., Sandri G., Listro R., Fagiani F., Collina S., Ferrari F. (2021). A composite nanosystem as a potential tool for the local treatment of glioblastoma: Chitosan-coated solid lipid nanoparticles embedded in electrospun nanofibers. Polymers.

[85] Shu X., Liu J., Mao L., Yuan F., Gao Y. (2024). Composite hydrogels filled with rhamnolipid-based nanoemulsion, nanostructured lipid carrier, or solid lipid nanoparticle: A comparative study on gel properties and the delivery of lutein. Food Hydrocoll..

[86] Gordillo-Galeano A., Mora-Huertas C.E. (2018). Solid lipid nanoparticles and nanostructured lipid carriers: A review emphasizing on particle structure and drug release. Eur. J. Pharm. Biopharm..

[87] Bunjes H. (2011). Structural properties of solid lipid based colloidal drug delivery systems. Curr. Opin. Colloid Interface Sci..

[88] Mohammed H.A., Khan R.A., Singh V., Yusuf M., Akhtar N., Sulaiman G.M., Albukhaty S., Abdellatif A.A.H., Khan M., Mohammed S.A.A. (2023). Solid lipid nanoparticles for targeted natural and synthetic drugs delivery in high-incidence cancers, and other diseases: Roles of preparation methods, lipid composition, transitional stability, and release profiles in nanocarriers’ development. Nanotechnol. Rev..

[89] Sakellari G., Zafeiri J., Batchelor H., Spyropoulos F. (2022). Solid lipid nanoparticles and nanostructured lipid carriers of dual functionality at emulsion interfaces. Part I: Pickering stabilization functionality. Colloids Surf. A Physicochem. Eng. Asp..

[90] Azhar Shekoufeh Bahari L., Hamishehkar H. (2016). The Impact of Variables on Particle Size of Solid Lipid Nanoparticles and Nanostructured Lipid Carriers; A Comparative Literature Review. Adv. Pharm. Bull..

[91] Guo X., Wang Y., Qin Y., Shen P., Peng Q. (2020). Structures, properties and application of alginic acid: A review. Int. J. Biol. Macromol..

[92] Taubner T., Marounek M., Synytsya A. (2017). Preparation and characterization of amidated derivatives of alginic acid. Int. J. Biol. Macromol..

[93] Kashif M., Ngaini Z., Harry A.V., Vekariya R.L., Ahmad A., Zuo Z., Sahari S.K., Hussain S., Khan Z.A., Alarifi A. (2020). An experimental and DFT study on novel dyes incorporated with natural dyes on titanium dioxide (TiO_2_) towards solar cell application. Appl. Phys. A.

[94] Ahmad A., Mubarak N.M., Jannat F.T., Ashfaq T., Santulli C., Rizwan M., Najda A., Bin-Jumah M., Abdel-Daim M.M., Hussain S. (2021). A Critical Review on the Synthesis of Natural Sodium Alginate Based Composite Materials: An Innovative Biological Polymer for Biomedical Delivery Applications. Processes.

[95] Yang J., Pan J. (2012). Hydrothermal synthesis of silver nanoparticles by sodium alginate and their applications in surface-enhanced Raman scattering and catalysis. Acta Mater..

[96] Jadach B., Świetlik W., Froelich A. (2022). Sodium Alginate as a Pharmaceutical Excipient: Novel Applications of a Well-known Polymer. J. Pharm. Sci..

[97] Karim A., Rehman A., Feng J., Noreen A., Assadpour E., Kharazmi M.S., Lianfu Z., Jafari S.M. (2022). Alginate-based nanocarriers for the delivery and controlled-release of bioactive compounds. Adv. Colloid Interface Sci..

[98] Abourehab M.A.S., Rajendran R.R., Singh A., Pramanik S., Shrivastav P., Ansari M.J., Manne R., Amaral L.S., Deepak A. (2022). Alginate as a Promising Biopolymer in Drug Delivery and Wound Healing: A Review of the State-of-the-Art. Int. J. Mol. Sci..

[99] Hegde V., Uthappa U.T., Altalhi T., Jung H., Han S.S., Kurkuri M.D. (2022). Alginate based polymeric systems for drug delivery, antibacterial/microbial, and wound dressing applications. Mater. Today Commun..

[100] Raus R.A., Nawawi W.M.F.W., Nasaruddin R.R. (2021). Alginate and alginate composites for biomedical applications. Asian J. Pharm. Sci..

[101] Venkatasubbu G.D., Ramasamy S., Ramakrishnan V., Kumar J. (2011). Hydroxyapatite-alginate nanocomposite as drug delivery matrix for sustained release of ciprofloxacin. J. Biomed. Nanotechnol..

[102] Soumia A., Adel M., Amina S., Bouhadjar B., Amal D., Farouk Z., Abdelkader B., Mohamed S. (2020). Fe_3_O_4_-alginate nanocomposite hydrogel beads material: One-pot preparation, release kinetics and antibacterial activity. Int. J. Biol. Macromol..

[103] Hamed S.F., Hashim A.F., Hamid H.A.A., Abd-Elsalam K.A., Golonka I., Musiał W., El-Sherbiny I.M. (2020). Edible alginate/chitosan-based nanocomposite microspheres as delivery vehicles of omega-3 rich oils. Carbohydr. Polym..

[104] Yu X., Wen T., Cao P., Shan L., Li L. (2019). Alginate-chitosan coated layered double hydroxide nanocomposites for enhanced oral vaccine delivery. J. Colloid Interface Sci..

[105] Malesu V.K., Sahoo D., Nayak P.L. (2011). Chitosan-Sodium Alginate Nanocomposites Blended with Cloisite 30B As a Novel Drug Delivery System for Anticancer Drug Curcumin. Int. J. Appl. Biol. Pharm..

[106] Nguyen-Ngo C., Willcox J.C., Lappas M. (2020). Anti-inflammatory effects of phenolic acids punicalagin and curcumin in human placenta and adipose tissue. Placenta.

[107] Cui J., Zhou J., Huang L., Jing J., Wang N., Wang L. (2019). Curcumin encapsulation and protection based on lysozyme nanoparticles. Food Sci. Nutr..

[108] Chegeni M., Rozbahani Z.S., Ghasemian M., Mehri M. (2020). Synthesis and application of the calcium alginate/SWCNT-Gl as a bio-nanocomposite for the curcumin delivery. Int. J. Biol. Macromol..

[109] El-Din H.M.N., Ibraheim D.M., Rabie A.G.M. (2023). Characterization and drug delivery characters of nanocomposite hydrogels based on gamma-radiation copolymerization of poly (vinyl pyrrolidone) (PVP)/sodium alginate (AG)/silver NPs. Int. J. Biol. Macromol..

[110] Kim J., Hlaing S.P., Lee J., Saparbayeva A., Kim S., Hwang D.S., Lee E.H., Yoon I., Yun H., Kim M. (2021). Exfoliated bentonite/alginate nanocomposite hydrogel enhances intestinal delivery of probiotics by resistance to gastric pH and on-demand disintegration. Carbohydr. Polym..

[111] Jindal R. (2021). RSM-CCD optimized microwave assisted synthesis of chitosan and sodium alginate based nanocomposite containing inclusion complexes of β-cyclodextrin and amlodipine besylate for sustained drug delivery systems. J. Drug Deliv. Sci. Technol..

[112] Fan Y., Wu W., Lei Y., Gaucher C., Pei S., Zhang J., Xia X. (2019). Edaravone-Loaded Alginate-Based Nanocomposite Hydrogel Accelerated Chronic Wound Healing in Diabetic Mice. Mar. Drugs.

[113] Agili F.A., Aly S.F.M. (2020). Physicochemical characterization and release properties of oral drug delivery: A pH-sensitive nanocomposite based on sodium alginate–pectin–tannic acid–silver. Polym. Polym. Compos..

[114] Shabanpour S., Shariati F.P., Khatibani A.B. (2022). Potential Alendronate Sodium drug carrier by preparation and characterization of sodium alginate cross-linked Montmorillonite. Braz. J. Pharm. Sci..

[115] Evelyna A., Astifanni T.K., Ruth I., Asri L., Purwasasmita B.S. (2019). Preparation of Nanocellulose-Alginate Nanocomposites for Chlorhexidine Digluconate Drug Carrier. IOP Conf. Ser..

[116] Lakkakula J., Roy A., Krishnamoorthy K., Alghamdi S., Almehmadi M., Gujarathi P., Pansare P., Allahyani M., Abdulaziz O., Velhal K. (2022). Alginate-Based Nanosystems for Therapeutic Applications. J. Nanomater..

[117] Iliescu R.I., Andronescu E., Ghitulica C.D., Voicu G., Ficai A., Hoteteu M. (2014). Montmorillonite–alginate nanocomposite as a drug delivery system—incorporation and in vitro release of irinotecan. Int. J. Pharm..

[118] Lei H., Xie M., Zhao Y., Zhang F., Xu Y., Xie J. (2016). Chitosan/sodium alginate modificated graphene oxide-based nanocomposite as a carrier for drug delivery. Ceram. Int..

[119] Iliescu R.I., Andronescu E., Ghiţulică C.D., Berger D., Ficai A. (2011). Montmorillonite-alginate nanocomposite beads as drug carrier for oral administration of carboplatin—preparation and characterization. U.P.B. Sci. Bull. Ser. B.

[120] Rosiak P., Latanska I., Paul P., Sujka W., Kolesinska B. (2021). Modification of Alginates to Modulate Their Physic-Chemical Properties and Obtain Biomaterials with Different Functional Properties. Molecules.

[121] Goh C.H., Heng P.W.S., Chan L.W. (2012). Alginates as a useful natural polymer for microencapsulation and therapeutic applications. Carbohydr. Polym..

[122] Kroneková Z., Pelach M., Mazancová P., Uhelská L., Treľová D., Rázga F., Némethová V., Szalai S., Chorvát D., McGarrigle J.J. (2018). Structural changes in alginate-based microspheres exposed to in vivo environment as revealed by confocal Raman microscopy. Sci. Rep..

[123] Abka-Khajouei R., Tounsi L., Shahabi N., Patel A.K., Abdelkafi S., Michaud P. (2022). Structures, Properties and Applications of Alginates. Mar. Drugs.

[124] Hasnain S., Jameel E., Mohanta B., Dhara A.K., Alkahtani S., Nayak A.K., Nayak A.K., Hasnain S. (2020). Chapter 1—Alginates: Sources, structure, and properties. Alginates in Drug Delivery.

[125] Wang B.-T., Hu S., Yu X.-Y., Jin L., Zhu Y.-J., Jin F.-J. (2020). Studies of Cellulose and Starch Utilization and the Regulatory Mechanisms of Related Enzymes in Fungi. Polymers.

[126] Moreira L.R., Filho E.X. (2016). Insights into the mechanism of enzymatic hydrolysis of xylan. Appl. Microbiol. Biotechnol..

[127] Zhang C., Keten S., Derome D., Carmeliet J. (2021). Hydrogen bonds dominated frictional stick-slip of cellulose nanocrystals. Carbohydr. Polym..

[128] Chang S., Weng Z., Zhang C., Jiang S., Duan G. (2023). Cellulose-Based Intelligent Responsive Materials: A Review. Polymers.

[129] Ningtyas K.R., Agassi T.N., Putri P.G. (2022). Utilization of Waste Cellulose Raw Material for Making Paper Pulp. IOP Conf. Ser..

[130] Mäkelä M., Rissanen M., Sixta H. (2021). Identification of cellulose textile fibers. Analyst.

[131] Chen C., Xi Y., Weng Y. (2022). Recent Advances in Cellulose-Based Hydrogels for Tissue Engineering Applications. Polymers.

[132] Joseph B., Sagarika V.K., Sabu C., Kalarikkal N., Sabu T. (2020). Cellulose nanocomposites: Fabrication and biomedical applications. J. Bioresour. Bioprod..

[133] Huo Y., Liu Y., Xia M., Du H., Lin Z., Li B., Liu H. (2022). Nanocellulose-Based Composite Materials Used in Drug Delivery Systems. Polymers.

[134] Prusty K., Swain S.K. (2019). Release of ciprofloxacin drugs by nano gold embedded cellulose grafted polyacrylamide hybrid nanocomposite hydrogels. Int. J. Biol. Macromol..

[135] Ji L., Zhang F., Zhu L., Jiang J. (2021). An in-situ fabrication of bamboo bacterial cellulose/sodium alginate nanocomposite hydrogels as carrier materials for controlled protein drug delivery. Int. J. Biol. Macromol..

[136] Rana A.K., Scarpa F., Thakur V.K. (2022). Cellulose/polyaniline hybrid nanocomposites: Design, fabrication, and emerging multidimensional applications. Ind. Crops Prod..

[137] Shahzadi I., Islam M., Saeed H., Shahzadi A., Haider J., Haider A., Imran M., Rathore H.A., Ul-Hamid A., Nabgan W. (2023). Facile synthesis of copolymerized cellulose grafted hydrogel doped calcium oxide nanocomposites with improved antioxidant activity for anti-arthritic and controlled release of doxorubicin for anti-cancer evaluation. Int. J. Biol. Macromol..

[138] Abukhadra M.R., Mohamed A.S., El-Sherbeeny A.M., Nadeem A., Ahmad S.F. (2020). Synthesis of exfoliate bentonite/cellulose nanocomposite as a delivery system for Oxaliplatin drug with enhanced loading and release properties; cytotoxicity and pharmacokinetic studies. Chem. Phys. Lett..

[139] Das S., Ghosh B., Sarkar K. (2022). Nanocellulose as sustainable biomaterials for drug delivery. Sens. Int..

[140] Patil T.V., Patel D.K., Dutta S.D., Ganguly K., Santra T.S., Lim K. (2022). Nanocellulose, a versatile platform: From the delivery of active molecules to tissue engineering applications. Bioact. Mater..

[141] Gholamali I., Yadollahi M. (2020). Doxorubicin-loaded carboxymethyl cellulose/Starch/ZnO nanocomposite hydrogel beads as an anticancer drug carrier agent. Int. J. Biol. Macromol..

[142] Sarkar C., Chowdhuri A.R., Kumar A., Laha D., Garai S., Chakraborty J., Sahu S.K. (2018). One pot synthesis of carbon dots decorated carboxymethyl cellulose-hydroxyapatite nanocomposite for drug delivery, tissue engineering and Fe^3+^ ion sensing. Carbohydr. Polym..

[143] Rakhshaei R., Namazi H., Hamishehkar H., Rahimi M. (2020). Graphene quantum dot cross-linked carboxymethyl cellulose nanocomposite hydrogel for pH-sensitive oral anticancer drug delivery with potential bioimaging properties. Int. J. Biol. Macromol..

[144] Rasoulzadeh M., Namazi H. (2017). Carboxymethyl cellulose/graphene oxide bio-nanocomposite hydrogel beads as anticancer drug carrier agent. Carbohydr. Polym..

[145] Salahuddin N., Gaber M., Mousa M., Elfiky M. (2023). Dopamine/Artesunate loaded polyhydroxybutyrate-g-cellulose- magnetite zinc oxide core shell nanocomposites: Synergistic antimicrobial and anticancer efficacy. Int. J. Biol. Macromol..

[146] Ghawanmeh A.A., Tan L.L., Ali G.A.M., Assiri M.A., Chong K.F. (2024). Optimization of carboxymethyl cellulose-gum Arab-based hydrogel beads for anticancer drugs delivery. J. Mol. Liq..

[147] Ostovar S., Pourmadadi M., Zaker M.A. (2023). Co-biopolymer of chitosan/carboxymethyl cellulose hydrogel improved by zinc oxide and graphene quantum dots nanoparticles as pH-sensitive nanocomposite for quercetin delivery to brain cancer treatment. Int. J. Biol. Macromol..

[148] Mandal B., Rameshbabu A.P., Dhara S., Pal S. (2017). Nanocomposite hydrogel derived from poly (methacrylic acid)/carboxymethyl cellulose/AuNPs: A potential transdermal drugs carrier. Polymer.

[149] Alsaaed F.A.T., El-Lateef H.M.A., Khalaf M.M., Mohamed I.M.A., Al-Omair M.A., Gouda M. (2022). Drug Delivery System Based on Carboxymethyl Cellulose Containing Metal-Organic Framework and Its Evaluation for Antibacterial Activity. Polymers.

[150] Moon R.J., Schueneman G.T., Simonsen J. (2016). Overview of cellulose nanomaterials, their capabilities and applications. JOM.

[151] Varghese R.T., Cherian R.M., Chirayil C.J., Antony T., Kargarzadeh H., Thomas S. (2023). Nanocellulose as an Avenue for Drug Delivery Applications: A Mini-Review. J. Compos. Sci..

[152] Chin S.F., Jimmy F.B., Pang S.C. (2018). Size controlled fabrication of cellulose nanoparticles for drug delivery applications. J. Drug Deliv. Sci. Technol..

[153] Mujtaba M., Negi A., King A.W.T., Zare M., Kuncova-Kallio J. (2023). Surface modifications of nanocellulose for drug delivery applications; a critical review. Curr. Opin. Biomed. Eng..

[154] Chua L., Lim P., Thoo Y., Neo Y., Tan T. (2023). Extraction and characterization of gelatin derived from acetic acid-treated black soldier fly larvae. Food Chem. Adv..

[155] Ahmad M.I., Li Y., Pan J., Liu F., Dai H., Fu Y., Huang T., Farooq S., Zhang H. (2024). Collagen and gelatin: Structure, properties, and applications in food industry. Int. J. Biol. Macromol..

[156] Rather J.A., Akhter N., Ashraf Q.S., Mir S.A., Makroo H.A., Darakshan Majid D., Barba F.J., Khaneghah A.M., Dar B.N. (2022). A comprehensive review on gelatin: Understanding impact of the sources, extraction methods, and modifications on potential packaging applications. Food Pack. Shelf Life.

[157] Luo Q., Hossen M.A., Zeng Y., Dai J., Li S., Qin W., Liu Y. (2022). Gelatin-based composite films and their application in food packaging: A review. J. Food Eng..

[158] Jiang X., Du Z., Zhang X., Zaman F., Song Z., Guan Y., Yu T., Huang Y. (2023). Gelatin-based anticancer drug delivery nanosystems: A mini review. Front. Bioeng. Biotechnol..

[159] Milano F., Masi A., Madaghiele M., Sannino A., Salvatore L., Gallo N. (2023). Current Trends in Gelatin-Based Drug Delivery Systems. Pharmaceutics.

[160] Dong Z., Meng X., Yang W., Zhang J., Sun P., Zhang H., Fang X., Wang D., Fan C. (2021). Progress of gelatin-based microspheres (GMSs) as delivery vehicles of drug and cell. Mater. Sci. Eng. C.

[161] Raj V., Prabha G. (2016). Synthesis, characterization and in vitro drug release of cisplatin loaded Cassava starch acetate–PEG/gelatin nanocomposites. J. Assoc. Arab Univ. Basic Appl. Sci..

[162] Najafabadi A.P., Pourmadadi M., Yazdian F., Rashedi H., Rahdar A., Díez-Pascual A.M. (2023). pH-sensitive ameliorated quercetin delivery using graphene oxide nanocarriers coated with potential anticancer gelatin-polyvinylpyrrolidone nanoemulsion with bitter almond oil. J. Drug Deliv. Sci. Technol..

[163] Mdlovu N.V., Juang R., Weng M., Lin K. (2023). Green synthesis and characterization of silicate nanostructures coated with Pluronic F127/gelatin for triggered drug delivery in tumor microenvironments. Int. J. Biol. Macromol..

[164] Ostovar S., Pourmadadi M., Shamsabadipour A., Mashayekh P. (2023). Nanocomposite of chitosan/gelatin/carbon quantum dots as a biocompatible and efficient nanocarrier for improving the Curcumin delivery restrictions to treat brain cancer. Int. J. Biol. Macromol..

[165] Kulshrestha A., Sharma S., Singh K., Kumar A. (2022). Magnetoresponsive biocomposite hydrogels comprising gelatin and valine based magnetic ionic liquid surfactant as controlled release nanocarrier for drug delivery. Mater. Adv..

[166] Moya-Lopez C., Juan A., Donizeti M., Valcarcel J., Vazquez J.A., Solano E., Chapron D., Bourson P., Bravo I., Alonso-Moreno C. (2022). Multifunctional PLA/Gelatin Bionanocomposites for Tailored Drug Delivery Systems. Pharmaceutics.

[167] Gheysoori P., Paydayesh A., Jafari M., Peidayesh H. (2023). Thermoresponsive nanocomposite hydrogels based on Gelatin/poly (N–isopropylacrylamide) (PNIPAM) for controlled drug delivery. Eur. Polym. J..

[168] Bhattacharyya S.K., Dule M., Paul R., Dash J., Anas M., Mandal T.K., Das P., Das N.C., Banerjee S. (2020). Carbon Dot Cross-Linked Gelatin Nanocomposite Hydrogel for pH-Sensing and pH-Responsive Drug Delivery. ACS Biomater. Sci. Eng..

[169] Bakravi A., Ahamadian Y., Hashemi H., Namazi H. (2018). Synthesis of gelatin-based biodegradable hydrogel nanocomposite and their application as drug delivery agent. Adv. Polym. Technol..

[170] Bora A., Sarmah D., Rather M.A., Mandal M., Karak N. (2024). Nanocomposite of starch, gelatin and itaconic acid-based biodegradable hydrogel and ZnO/cellulose nanofiber: A pH-sensitive sustained drug delivery vehicle. Int. J. Biol. Macromol..

[171] Tanwar A., Kalode P., Roshni V., Prema B.K., Doshi P., Ottoor D. (2023). Influence of nanofillers (Ag NPs and C. dots) on the controlled drug release profile of gelatin-grafted-polyacrylamide hydrogel: An in vitro study. Mater. Today Commun..

[172] Li C., Li F., Wang K., Wang Q., Liu H., Sun X., Xie D. (2023). Synthesis, characterizations, and release mechanisms of carboxymethyl chitosan-graphene oxide-gelatin composite hydrogel for controlled delivery of drug. Inorg. Chem. Commun..

[173] Singh H., Yadav I., Sheikh W.M., Dan A., Darban Z., Shah S.A., Mishra N.C., Shahabuddin S., Hassan S., Bashir S.M. (2023). Dual cross-linked gellan gum/gelatin-based multifunctional nanocomposite hydrogel scaffold for full-thickness wound healing. Int. J. Biol. Macromol..

[174] Hezari S., Olad A., Dilmaghani A. (2022). Modified gelatin/iron- based metal-organic framework nanocomposite hydrogel as wound dressing: Synthesis, antibacterial activity, and Camellia sinensis release. Int. J. Biol. Macromol..

[175] Alarçin E., Dokgöz A.B., Akgüner Z.P., Seki H.K., Bal-Öztürk A. (2022). Gelatin methacryloyl/nanosilicate nanocomposite hydrogels encapsulating dexamethasone with a tunable crosslinking density for bone repair. J. Drug Deliv. Sci. Technol..

[176] Rahmani S., Olad A., Rahmani Z. (2023). Preparation of self-healable nanocomposite hydrogel based on Gum Arabic/gelatin and graphene oxide: Study of drug delivery behavior. Polym. Bull..

[177] Jaberifard F., Arsalani N., Ghorbani M., Mostafavi H. (2022). Incorporating halloysite nanotube/carvedilol nanohybrids into gelatin microsphere as a novel oral pH-sensitive drug delivery system. Colloids Surf. A.

[178] Mathew S.A., Arumainathan S. (2022). Crosslinked Chitosan–Gelatin Biocompatible Nanocomposite as a Neuro Drug Carrier. ACS Omega.

[179] Baydin T., Aarstad O.A., Dille M.J., Hattrem M.N., Draget K.I. (2022). Long-term storage stability of type A and type B gelatin gels: The effect of Bloom strength and co-solutes. Food Hydrocoll..

[180] Dranca I., Vyazovkin S. (2009). Thermal stability of gelatin gels: Effect of preparation conditions on the activation energy barrier to melting. Polymer.

[181] Tan Y., Zi Y., Peng J., Shi C., Zheng Y., Zhong J. (2023). Gelatin as a bioactive nanodelivery system for functional food applications. Food Chem..

[182] Singh S., Manikandan R., Singh S. (2000). Stability testing for gelatin-based formulations: Rapidly evaluating the possibility of a reduction in dissolution rates. Pharm. Technol..

[183] Foox M., Zilberman M. (2015). Drug delivery from gelatin-based systems. Expert Opin. Drug Deliv..

[184] Alipal J., Mohd Pu’ad N.A.S., Lee T.C., Nayan N.H.M., Sahari N., Basri H., Idris M.I., Abdullah H.Z. (2021). A review of gelatin: Properties, sources, process, applications, and commercialisation. Mater. Today Proc..

[185] Hutapea T.P.H., Madurani K.A., Syahputra M.Y., Hudha M.N., Asriana A.N., Kurniawan F. (2023). Albumin: Source, preparation, determination, applications, and prospects. J. Sci. Adv. Mater. Devices.

[186] Tiwari R., Sethiya N.K., Gulbake A.S., Mehra N.K., Murty U.S.N., Gulbake A. (2021). A review on albumin as a biomaterial for ocular drug delivery. Int. J. Biol. Macromol..

[187] Pompili E., Zaccherini G., Baldassarre M., Iannone G., Caraceni P. (2023). Albumin administration in internal medicine: A journey between effectiveness and futility. Eur. J. Intern. Med..

[188] Mishra V., Heath R.J. (2021). Structural and Biochemical Features of Human Serum Albumin Essential for Eukaryotic Cell Culture. Int. J. Mol. Sci..

[189] Wouw J., Joles J.A. (2022). Albumin is an interface between blood plasma and cell membrane, and not just a sponge. Clin Kidney J..

[190] Xu X., Jinyu Hu J., Xue H., Hu Y., Liu Y., Lin G., Liu L., Xu R. (2023). Applications of human and bovine serum albumins in biomedical engineering: A review. Int. J. Biol. Macromol..

[191] Spada A., Emami J., Tuszynski J.A., Lavasanifar A. (2021). The Uniqueness of Albumin as a Carrier in Nanodrug Delivery. Mol. Pharm..

[192] Wang Y., Iqbal H., Ur-Rehman U., Zhai L., Yuan Z., Razzaq A., Lv M., Wei H., Ning X., Xin J. (2023). Albumin-based nanodevices for breast cancer diagnosis and therapy. J. Drug Deliv. Sci. Technol..

[193] Hoogenboezem E.N., Duvall C.L. (2018). Harnessing Albumin as a Carrier for Cancer Therapies. Adv. Drug Deliv. Rev..

[194] Ma N., Liu J., He W., Li Z., Luan Y., Song Y., Garg S. (2017). Folic acid-grafted bovine serum albumin decorated graphene oxide: An efficient drug carrier for targeted cancer therapy. J. Colloid Interface Sci..

[195] Akbal O., Vural T., Malekghasemi S., Bozdoğan B., Denkbaş E.B. (2018). Saponin loaded montmorillonite-human serum albumin nanocomposites as drug delivery system in colorectal cancer therapy. Appl. Clay Sci..

[196] Yu X., Xu Z., Wang X., Xu Q., Chen J. (2020). Bactrian camel serum albumins-based nanocomposite as versatile biocargo for drug delivery, biocatalysis and detection of hydrogen peroxide. Mater. Sci. Eng. C.

[197] Elgohary M.M., Helmy M.W., Abdelfattah E.A., Ragab D.M., Mortada S.M., Fang J., Elzoghby A.O. (2018). Targeting sialic acid residues on lung cancer cells by inhalable boronic acid-decorated albumin nanocomposites for combined chemo/herbal therapy. J. Control. Release.

[198] Chen Z., Hong G., Liu Z., Yang D., Kankala R.K., Wu W. (2020). Synergistic antitumor efficacy of doxorubicin and gambogic acid-encapsulated albumin nanocomposites. Colloids Surf. B.

[199] Liu Z., Wang X., Zhang C., Lin K., Yang J., Yi Zhang Y., Hao J., Tian F. (2024). Folic acid-coupled bovine serum albumin-modified magnetic nanocomposites from quantum-sized Fe_3_O_4_ and layered double hydroxide for actively targeted delivery of 5-fluorouracil. Int. J. Biol. Macromol..

[200] Xinyu Y., Adilijiang X., Qilan X., Azhati Z., Yiyan S., Ling C., Jin C. (2020). GSH-responsive curcumin/doxorubicin encapsulated Bactrian camel serum albumin nanocomposites with synergistic effect against lung cancer cells. J. Biomed. Sci..

[201] Bardania H., Jafari F., Baneshi M., Mahmoudi R., Ardakani M.T., Safari F., Barmak M.J. (2023). Folic Acid-Functionalized Albumin/Graphene Oxide Nanocomposite to Simultaneously Deliver Curcumin and 5-Fluorouracil into Human Colorectal Cancer Cells: An In Vitro Study. Biomed Res. Int..

[202] Jalali E.S., Shojaosadati S.A., Hamedi S. (2023). Green synthesis of bovine serum albumin/oxidized gum Arabic nanocomposite as pH-responsive carrier for controlled release of piperine and the molecular docking study. Int. J. Biol. Macromol..

[203] Jing W., Jiang M., Fu X., Yang J., Chen L., Leng F., Xu P., Huang W., Yang C.Y.Z. (2022). Self-assembly drug-albumin nanocomposites for nonalcoholic fatty liver disease treatment. Int. J. Biol. Macromol..

[204] Madeira P.P., Rocha I.L.D., Rosa M.E., Freire M.G., Coutinho J.A.P. (2022). On the aggregation of bovine serum albumin. J. Mol. Liq..

[205] Karimi M., Bahrami S., Ravari S.B., Zangabad P.S., Mirshekari H., Bozorgomid M., Shahreza S., Sori M., Hamblin M.R. (2016). Albumin nanostructures as advanced drug delivery systems. Expert Opin. Drug Deliv..

[206] Larsen M.T., Kuhlmann M., Hvam M.L., Howard K.A. (2016). Albumin-based drug delivery: Harnessing nature to cure disease. Mol. Cell. Ther..

